# Balancing Stealth and Targetability: cCPP‐ and PEG‐Modified Liposomes for the Targeted Delivery of Anti‐HER2 Nanobodies

**DOI:** 10.1002/advs.75646

**Published:** 2026-05-11

**Authors:** Giulia Pander, Maria del Pilar Palacios Cisneros, Clara Certa, Megan Stierli, Katharina Beck, Nicolas Färber, Lisa Blank, Fiona Tanner, Eric Mühlberg, Sabrina Wohlfart, Christian Kleist, Jörg Huwyler, Gert Fricker, Walter Mier, Philipp Uhl

**Affiliations:** ^1^ Department of Pharmaceutical Technology Institute of Pharmacy and Molecular Biotechnology Heidelberg University Heidelberg Germany; ^2^ Department of Nuclear Medicine Heidelberg University Hospital Heidelberg Germany; ^3^ Department of Pharmaceutical Technology University of Basel Basel Switzerland; ^4^ Lipospec GmbH Augsburg Germany; ^5^ Physiology, Institute of Theoretical Medicine University of Augsburg, Universitätsstraße 2 Augsburg Germany

**Keywords:** HER2, liposomes, nanobodies, pharmacokinetics, rats, zebrafish

## Abstract

Nanobodies (Nbs) are considered promising antibody fragments for overcoming limitations in precision oncology due to their high specificity and deep tissue penetration. However, their therapeutic potential remains limited by their rapid renal clearance. In this study, anti‐HER2 Nb‐loaded liposomes with dual functionalization combining polyethylene glycol 2000 (PEG) and cyclic cell‐penetrating peptides are developed to ameliorate their pharmacokinetic behavior while retaining binding specificity. Liposomal formulations with high encapsulation efficiencies are produced with dual centrifugation. Biophysical characterization reveals that PEGylation effectively mitigates cCPP‐induced membrane destabilization, ensuring structural integrity. In vitro assays confirm that, despite the steric shielding by PEG, the encapsulated Nbs retain their functionality and specific binding to HER2‐overexpressing cells. In vivo studies in zebrafish larvae demonstrate excellent biocompatibility and lack of immunogenicity. Crucially, liposomal encapsulation significantly modulates the pharmacokinetic profile of Nbs in rats, reducing renal accumulation compared to free Nbs. This study presents a robust liposomal platform that successfully balances the stealth properties of PEG with the functional benefits of cCPPs. Consequently, this platform offers an effective strategy to enhance the therapeutic window of low‐molecular‐weight biologics.

## Graphical abstract

This study addresses the rapid renal clearance of anti‐HER2 nanobodies by developing specialized liposomal carriers. It details the formulation of dual modified liposomes, demonstrating how PEGylation preserves structural integrity and cell‐penetrating peptides enhance cellular interaction. Findings confirm maintained binding specificity in vitro, biocompatibility in zebrafish, and improved pharmacokinetic profiles in rats, offering a robust platform for enhancing low‐molecular‐weight biologics.

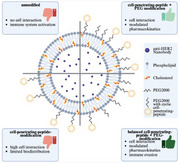

## 1 Introduction

Nanobodies (Nbs) represent an emerging class of antibody fragments that combine the beneficial properties of full‐length monoclonal antibodies (mAbs), such as high specificity and affinity, with advantageous physicochemical properties [1]. Due to their smaller size (∼15–20 kDa), Nbs exhibit superior tissue penetration, increased stability against pH variations, and enzymatic degradation [2, 3]. However, their low molecular weight falls below the renal filtration threshold, rendering them susceptible to rapid blood clearance and significant accumulation in the kidneys. This limits their plasma half‐life and therapeutic efficacy, particularly in oncological applications where sustained exposure is critical [4, 5]. Until now, only a handful of Nbs have reached commercial approval, while a large number is currently in clinical development [6]. To overcome the pharmacokinetic limitations, we investigated the encapsulation of Nbs into liposomal drug‐delivery systems by using dual centrifugation (DC) to increase the hydrodynamic radius, thereby reducing renal uptake and modulating the biodistribution profile. For this study, we utilized a recombinant anti‐human epidermal growth factor receptor 2 (HER2) Nb. HER2 is overexpressed in various solid tumor entities, such as breast, gastric, bladder, salivary gland, cervical, ovarian, endometrial, colorectal, lung and biliary tract cancers, often accounting for aggressive tumor progression and poor outcome [7, 8]. Due to its pan‐tumor expression profile, with overexpression observed in approximately 15%–30% of breast cancers and about 10%–30% of gastric/gastroesophageal cancers, HER2 represents a prime candidate for targeted therapies [9, 10, 11]. Moreover, due to its well‐characterized biology, HER2 serves as an established benchmark target for the evaluation of novel tumor‐targeting nanocarriers [12].

Liposomes are versatile nanocarriers that can be tailored to specific therapeutic needs, such as reducing systemic toxicity, prolonging circulation time, and active targeting [13, 14]. To our knowledge, up to date, no data for liposomal encapsulated Nbs are available. Therefore, to enhance circulation time and reduce immunogenicity, we chose PEGylation as a shielding modification, a strategy well‐known from marketed products such as Doxil [13]. Surface modification with polyethylene glycol (PEG) is widely employed to create ‘stealth’ liposomes that prevent opsonization and subsequent recognition by the reticuloendothelial system, thereby significantly extending blood circulation times [15]. Furthermore, to counterbalance the potentially reduced cellular uptake often associated with PEGylation [16], the liposomes were surface‐modified with cyclic cell‐penetrating peptides (cCPP). While this modification strategy has previously been shown by our group to enhance mucosal penetration after oral administration [17], in this study, this modification aims to restore and enhance cellular interaction and receptor binding efficacy.

Here, we demonstrate the production of an anti‐HER2 Nb that specifically targets HER2‐overexpressing breast cancer cells (SKBr3) while low‐expressing cells (MCF‐7) do not exhibit receptor binding. We successfully encapsulated the Nb into dual functionalized liposomes that contained 5 mol% PEG2000 and 0.5 or 1 mol% cCPP, preserving and even outperforming its bioactivity. The formulations were tested with respect to in vitro cytotoxicity and binding assays and screened for circulation and immune evasion in a zebrafish larvae model. Finally, biodistribution and pharmacokinetic profiles were quantified with radiolabeled Nbs in healthy Wistar rats to establish a baseline for these novel formulations.

With this approach, we developed dual surface‐modified liposomes as a carrier system for an anti‐HER2 Nb that significantly reduces renal clearance and shifts its biodistribution toward the liver. This study provides a proof‐of‐concept for the successful encapsulation and biological activity retention of low‐molecular‐weight biologics.

## 2 Results

### 2.1 Anti‐HER2 Nanobody Production and Purification in *E. coli*

**FIGURE 1:**
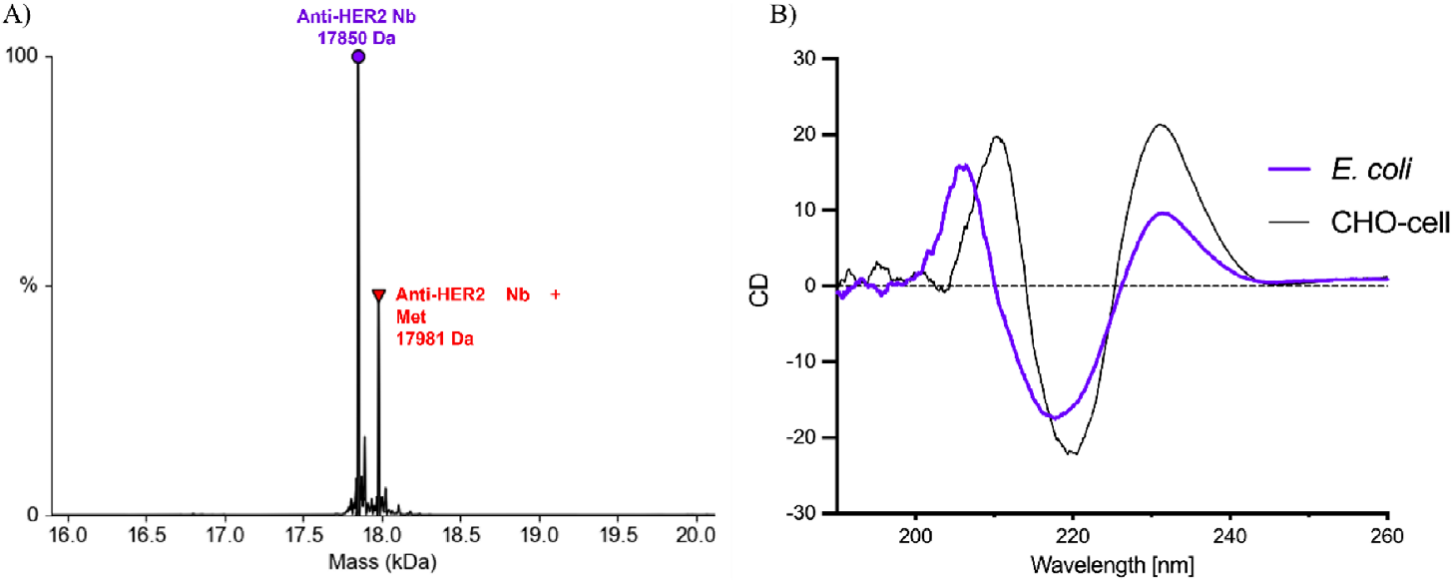
(A) Intact mass analysis of the purified nanobody confirming identity and high purity. The deconvoluted mass spectrum (ESI‐MS) displays a primary peak at 17850 Da, which is consistent with the theoretical molecular weight of the anti‐HER2 Nb (calc. MW: 17852.37 Da). The secondary minor peak at 17981 Da (+131 Da) corresponds to the species with a retained N‐terminal methionine. (B) Far‐UV CD spectroscopy confirms correct secondary structure folding. CD spectrum of the refolded *E. coli*‐expressed Nb (lilac) and CD spectrum of a CHO‐expressed control (black). Both proteins exhibit a characteristic spectral profile with a distinct minimum at ∼216 nm, indicative of the predominant β‐sheet structure typical for immunoglobulin domains. The consistency between the spectral signatures confirms successful refolding of the bacterial product.

The anti‐HER2 Nb was expressed and purified, yielding 110 mg (SD = 8.16) of highly pure protein per liter of bacterial culture. Its purity was confirmed by SDS‐PAGE (Figure S1), showing a single band consistent with its expected molecular weight (∼17 kDa). Purity was further validated by mass spectrometry (Figure 1), which revealed a main peak at 17850 Da. The second, smaller peak with a mass difference of 131 g/mol fits the molecular weight of the amino acid methionine, indicating an N‐terminal methionine‐prolonged Nb.

Structural analysis via circular dichroism (CD) spectroscopy (Figure 1B) confirmed the correct folding of the refolded Nb. The CD spectrum showed a strong correspondence to that of a CHO cell‐expressed analogue, with characteristic negative and positive bands at 216 nm and 207 nm, respectively. These features indicate that the protein is dominated by β‐sheets and β‐turns. The purified Nb has a theoretical isoelectric point (pI) of 6.2 and was therefore stored at 4 °C in TRIS buffer (pH 8) and rebuffered to PBS for subsequent experiments. The amino acid sequences of both Nbs are provided in Section S1, together with confocal analysis (Figure S2) showing a similar binding pattern and strength for both Nbs.

### 2.2 Nanobody Binding to HER2 Overexpressing Cells

The functionality of the anti‐HER2 Nb was evaluated by assessing its binding to the HER2 receptor on cell lines with varying expression levels. The breast cancer cell line SKBr3, which overexpresses the HER2 receptor (referred to as HER2^+^), was used as the target cell line. Conversely, MCF‐7 cells, which express HER2 only marginally (HER2–), served as a negative control. The degree of fluorescence labeling was tuned to 2–2.5 dye molecules per protein for both the Nb and trastuzumab with a treatment concentration of 5 µm for confocal analysis and flow cytometry.

#### 2.2.1 Confocal Microscopy Analysis

**FIGURE 2:**
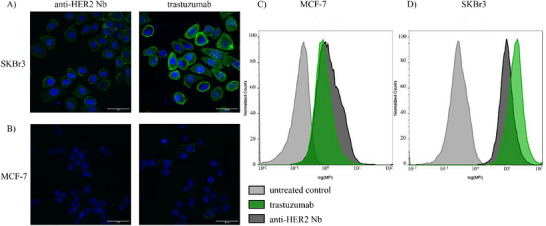
Confocal microscopy and flow cytometry analysis confirm specific binding of the anti‐HER2 nanobody to HER2^+^ cells. (A) Analysis of HER2^+^ SKBR3 cells incubated with equimolar concentration (5 µm) Nb or trastuzumab (positive control), with a comparable degree of labeling. Both the Nb and trastuzumab display a distinct membranous fluorescence pattern (green), confirming specific receptor recognition. While trastuzumab exhibits a higher overall signal intensity, the localization profile is identical to the Nb. (B) Analysis of HER2– MCF‐7 control cells. Neither the Nb nor trastuzumab showed detectable binding, confirming the specificity of the interaction. Microscopy details: Green FITC channel: Atto488‐labeled protein; Blue DAPI channel: Hoechst‐stained nuclei. Scale bars = 50 µm (C+D). Flow cytometry analysis of HER2 binding specificity. Histograms of MCF‐7 (C) and SKBr3 (D) cells incubated with equimolar concentration (5 µm) trastuzumab (green) or anti‐HER2 Nb (dark grey) compared to untreated control cells (light grey). Consistent with confocal microscopy findings, no significant binding is observed on MCF‐7 cells, where signals overlap with the control. In contrast, a distinct shift in fluorescence intensity is visible for both the Nb and trastuzumab on SKBr3 cells, confirming specific receptor recognition.

Confocal microscopy was employed to visualize the binding of the anti‐HER2 Nb to these cell lines. The images (Figure 2A) show a distinct binding pattern: the Nb strongly binds to the surface of the HER2^+^ SKBr3 cells as demonstrated by the green FITC signal, but exhibits no detectable binding to the HER2– MCF‐7 cells. For validation, fluorescently labeled trastuzumab was used as a positive control in the same experimental setup. Confocal images of trastuzumab displayed a comparable binding pattern, confirming the specific binding affinity of the anti‐HER2 Nb.

#### 2.2.2 Flow Cytometry Analysis

To quantify the binding specificity, flow cytometry was performed on HER2^+^ SKBr3 and HER2– MCF‐7 cells. Trastuzumab was included as a benchmark, with untreated cells serving as the negative control. The histograms in Figure 2B display a distinct separation between treated and untreated populations on SKBr3 cells, whereas signals on MCF‐7 cells remained near baseline levels.

To compare the specificity, the ratio of the background‐corrected median fluorescence intensity on target versus non‐target cells was calculated (Table S1 and Equation S1). The anti‐HER2 Nb exhibited an 8.9‐fold higher specific binding to SKBr3 cells compared to MCF‐7 cells. Trastuzumab displayed a 26.8‐fold higher specific binding ratio.

### 2.3 Nb‐Loaded Liposomes can be Prepared With Dual Centrifugation and Show High Encapsulation Efficiency

**FIGURE 3:**
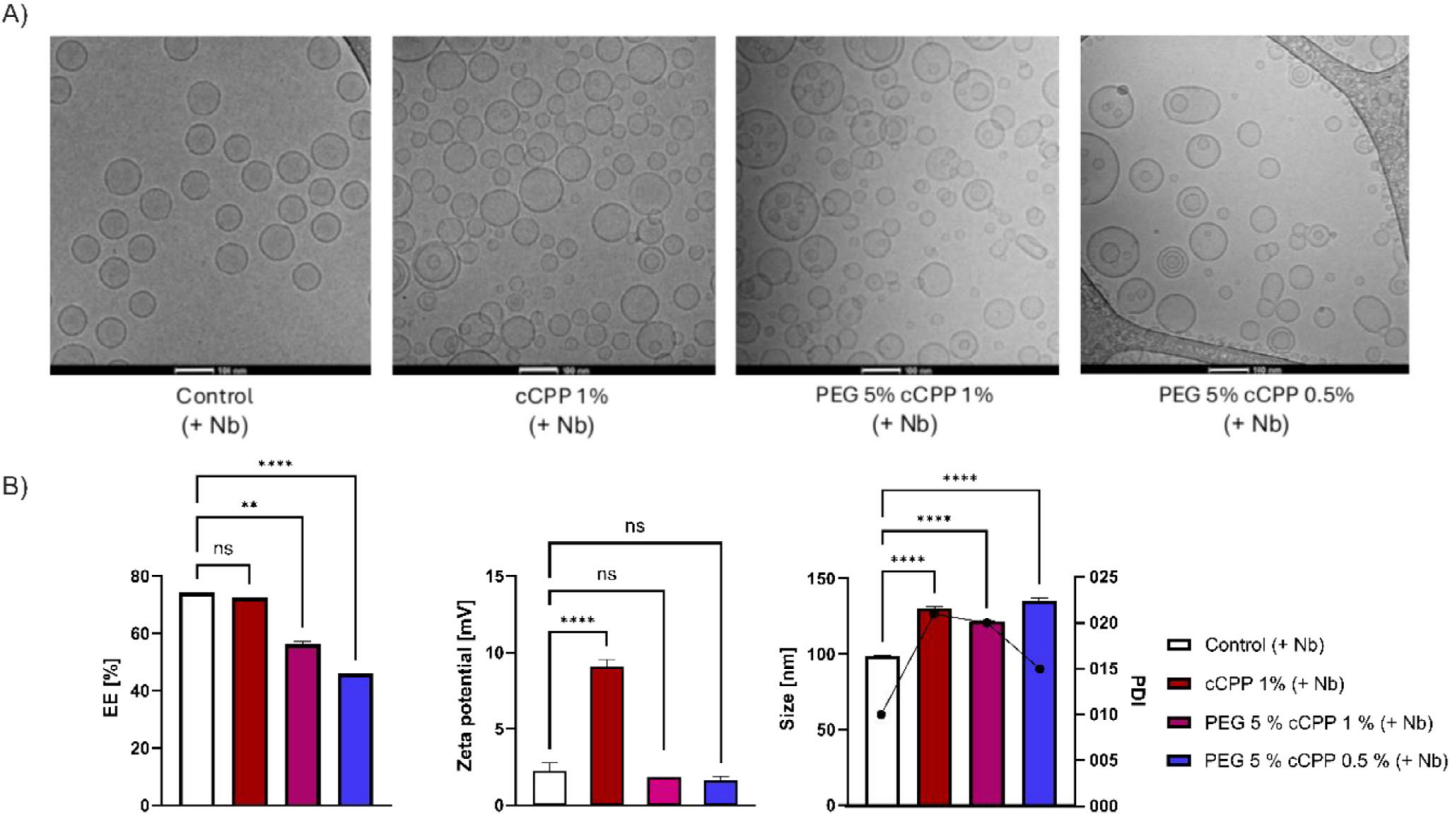
Physicochemical characterization and morphology of nanobody‐loaded liposomes. (A) Representative cryogenic transmission electron microscopy (Cryo‐TEM) micrographs of the four liposomal formulations. Images confirm the formation of spherical, unilamellar vesicles (SUVs) with a uniform size distribution. Scale bars = 100 nm. (B) Quantitative analysis of Encapsulation Efficiency (EE), zeta potential, and hydrodynamic diameter (Z‐average, Size) with superimposed polydispersity index (PDI). While the cCPP 1% formulation showed a significantly increased zeta potential, the PEG 5% cCPP 1% and PEG 5% cCPP 0.5% formulations exhibited values comparable to the control, confirming effective charge shielding by the PEG layer. All formulations showed a PDI < 0.25. Data information: Data are presented as mean ± SD from n = 3 independent production batches. Statistical significance was determined by one‐way ANOVA followed by Dunnett's multiple comparison test (^****^ *p* < 0.0001, ^**^ *p* < 0.01, *ns* = *p* > 0.05).

Four different liposomal formulations based on a control composition of 90 mol% soy phosphatidylcholine (SPC) and 10 mol% cholesterol were prepared. In addition to the unmodified control, one 1 mol% cCPP‐modified variant (cCPP 1%) and two dual‐functionalized formulations containing 5 mol% PEG with either 0.5 or 1 mol% cCPP (denoted as PEG 5% cCPP 0.5% and PEG 5% cCPP 1%, respectively) were produced. As presented in Figure 3, particle size analysis revealed that all liposomal formulations showed homogeneity, indicated by a low polydispersity index (PDI < 0.25). Although the hydrodynamic diameter increased slightly for the PEG 5% cCPP 1% (135.2 ± 1.6 nm), PEG 5% cCPP 0.5% (121.5 ± 0.2 nm), and cCPP 1% formulations (130.0 ± 1.0 nm), compared to the control formulation (98.64 ± 0.7), all vesicles remained within a size range suitable for intravenous application.

Regarding surface charge, the successful incorporation of the cCPP‐lipid was confirmed by a significant increase in zeta potential (*p* < 0.0001) for the cCPP 1% formulation compared to the unmodified control. Interestingly, the PEG 5% cCPP 1% and PEG 5% cCPP 0.5% formulations showed no significant shift in zeta potential compared to the control. This indicates that the PEG layer effectively masks the positive charge of the peptides (“shielding effect”).

Encapsulation efficiency (EE) was analyzed for all formulations using radioactive tracing. Unmodified control liposomes served as a baseline, exhibiting a high EE of 74.21% ± 0.01%. The incorporation of 1 mol% cCPP‐lipid did not significantly alter the encapsulation efficiency (72.35% ± 0.02%). However, the addition of PEG‐lipids resulted in a significant decrease in EE. The PEG 5% cCPP 1% formulation showed an EE of 55.49% ± 0.03%. This reduction was even more pronounced in the PEG 5% cCPP 0.5% formulation, dropping to 45.88% ± 0.61%.

These quantitative findings were corroborated by cryogenic transmission electron microscopy (Cryo‐TEM), which revealed a uniform population of predominantly small, unilamellar vesicles (SUVs) for all liposomal groups, with no significant morphological alterations following surface modification.

### 2.4 Laurdan Fluorescence Spectroscopy

**FIGURE 4:**
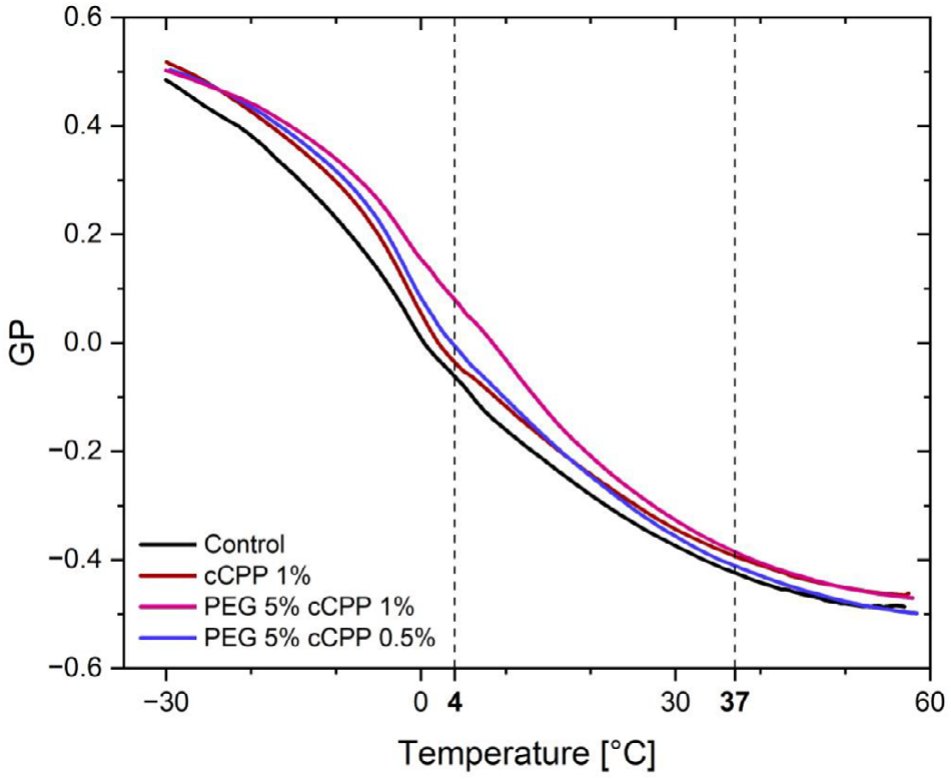
Laurdan generalized polarization (GP) as a function of temperature during the first heating scan. Liposomal formulations contained 0.5 mol% Laurdan (scan rate: 0.5 K min^−^^1^). Buffer: PBS, 4°C (storage temperature) and 37°C (physiological temperature) are indicated. All surface‐modified liposomes exhibited slightly higher GP values compared to the control formulation, with the PEG 5% cCPP 1% (pink) displaying the most pronounced elevation. The displayed data points are averages of 9 consecutive measurements, using a moving average filter.

The influence of cCPP‐lipid and PEG‐lipid on lipid membrane properties is reflected in the generalized polarization (GP) values obtained from the emission spectra of membrane‐embedded Laurdan. Incorporation of Laurdan did not significantly change liposomal characteristics (Figure S3). GP values serve as an indicator of lipid order, membrane fluidity, and packing density [18, 19, 20]. Figure 4 shows GP as a function of temperature for the first heating (up) scan over a range of −30°C to 60°C. Overall, GP values decreased monotonically with increasing temperature, reflecting a temperature‐dependent increase in membrane fluidity. A broad thermotropic phase transition, with the most pronounced change in slope around 0°C to15°C was observed.

All modified formulations (addition of PEG‐lipid and/or cCPP‐lipid) exhibited higher GP values than the control liposomes, indicating increased lipid order. The most pronounced increase was observed for PEG 5% cCPP 1% formulation (pink curve). Differences between formulations were most evident at low to intermediate temperatures and attenuated at higher temperatures. At physiological temperature (37°C), the variance was diminished. The temperature range of the thermotropic phase transition and the overall profile of the GP curves remained highly comparable. The addition of PEG‐lipids did not further broaden the phase transition.

**FIGURE 5:**
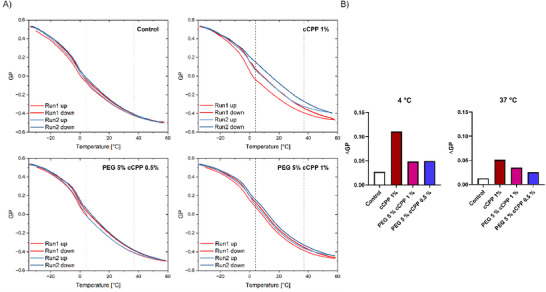
(A) Laurdan generalized polarization (GP) as a function of temperature showing the first four consecutive temperature scans (i.e., two heating scans and two cooling scans). Panels display: Control liposomes (top left), cCPP 1% (top right), PEG 5% cCPP 0.5% (bottom left), and PEG 5% cCPP 1% (bottom right). Scan rate: 0.5 K min^−^^1^. Buffer: PBS; Curves do not superimpose over consecutive cycles; all other formulations show reversible scans. The displayed data points are averages of 9 consecutive measurements, using a moving average filter. (B) Differences in Laurdan GP between the first heating and first cooling scans, expressed as ΔGP at 4°C (storage temperature) and 37°C (physiological temperature) for the indicated liposomal formulations. The cCPP 1% formulation displayed hysteresis, while the inclusion of PEG in the formulations attenuated this effect.

Differences among the liposome formulations became apparent when considering the subsequent cooling scan. Figure 5A shows the GP curves obtained from a series of heating and cooling scans.

Figure 5B summarizes the corresponding scan‐to‐scan differences as ΔGP at 4°C (storage temperature) and 37°C (physiological temperature). The control formulation exhibited a reversible thermal profile with negligible differences between heating and cooling scans, indicating a reversible thermotropic phase transition. In contrast, the cCPP 1% formulation displayed a pronounced hysteresis. The formulations containing PEG‐lipids exhibited reduced hysteresis in comparison with cCPP 1% alone. Again, for all formulations, ΔGP between heating and cooling scans was more pronounced at 4°C than at 37°C.

In addition, the results depicted in Figure 5 reveal a further difference associated with the incorporation of cCPP. While the control formulation showed highly reproducible GP profiles across repeated cycles, the second heating scan of the cCPP 1% formulation was shifted to higher values relative to the first one. This shift is particularly evident when comparing the terminal GP values at 60°C. Notably, inclusion of PEG‐lipid minored this effect, and no apparent shift was observed for PEG 5% cCPP 0.5%.

As the experimental conditions indicated potential instability for cCPP‐containing formulations triggered by thermal stress, the stability of the cCPP 1% formulation was further evaluated via a 24‐h time‐scan at 37°C (Figure S4). Crucially, the GP values remained constant over the 24‐h period, confirming that the formulation maintains its stability at physiological temperature.

### 2.5 Biocompatibility of Liposomal Nanobody Formulations

#### 2.5.1 Cytotoxicity

**FIGURE 6:**
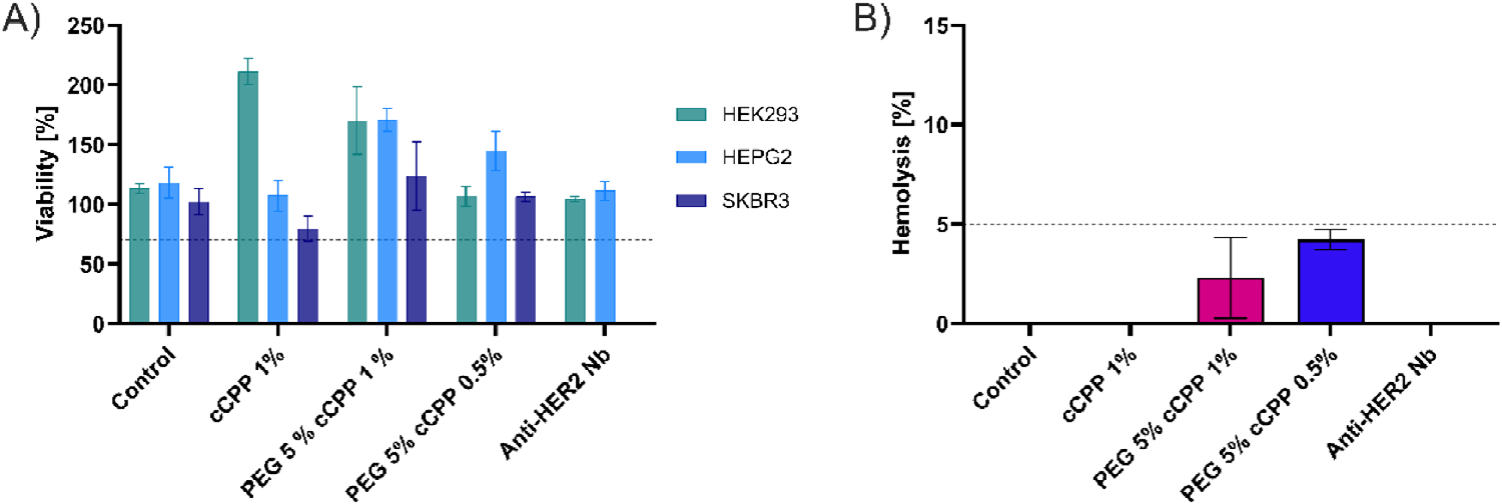
Cytotoxicity and hemolysis assay confirm the biocompatibility of the liposomal carrier systems. (A) Cell viability of HEK293, HepG2, and SKBr3 cells was assessed after incubation with empty liposomal formulations (33.3 mm lipid). The free Nb (50 µm) was included in the analysis for HEK293 and HepG2 cells. Viability was normalized to the untreated positive control (set to 100%). All tested substances showed cell viability above the 70% threshold, classifying them as non‐cytotoxic according to ISO 10993–5. Data are presented as mean ± SD from n = 3 independent biological replicates. (B) Hemolytic activity was assessed after incubation of washed erythrocytes with empty liposomal formulations (12.5 mm) and Nb (25 µm), respectively. Hemolysis was normalized to a Triton X‐100 control (set to 100%). All tested substances showed hemolytic activity below the acceptance criterion of 5%. Data are presented as mean ± SD from n = 3 independent biological replicates.

The kidneys and liver represent the primary organs for drug metabolism and excretion and are commonly used to assess off‐target cytotoxicity. Accordingly, the human cell lines HEK293 and HepG2 served as kidney and liver tissue models, respectively, in in vitro cytotoxicity assays to determine the safety profile of the Nb and all empty liposomal formulations. All empty liposomal formulations were additionally tested on SKBr3 cells to ensure that cytotoxic findings on target cells can only be attributed to the Nb itself. To guarantee the biocompatibility of in vitro findings for potential in vivo applications, all liposomal formulations were tested at their highest applicable concentration, which equaled the lipid concentration after liposomal purification (33.3 mM). Untreated cells were used as a 100% cell viability control. According to ISO 10993–5 [21], substances with viability values above 70% are regarded as non‐cytotoxic. This threshold is indicated by the dashed line in Figure 6A. For all liposomal formulations, the results showed no detectable cytotoxicity on either HepG2, HEK293, or SKBr3 cells, indicating a favorable safety profile at the highest intended dose. Similarly, the free Nb was tested at the highest concentration of 25 µm to ensure the absence of off‐target effects. No apparent cytotoxicity was observed on either liver or kidney cells for the free Nb. These findings suggest that both the Nb and all liposomal formulations are well‐tolerated by the metabolizing organs, which is a vital prerequisite for further in vivo investigation.

#### 2.5.2 Hemolysis

As depicted in Figure 6B, the hemolytic potential of the highest tested concentrations of all liposomal formulations and the free anti‐HER2 Nb was evaluated. The acceptance criterion or classifying a substance as non‐hemolytic is conventionally set at ≤5% hemolysis relative to the positive control [22].

All tested formulations, including the free Nb and the highest concentration (12.5 mm lipid/50 µm Nb) of all liposomal formulations, demonstrated a percentage of hemolysis below 5%. This conclusively shows that all tested formulations are considered non‐hemolytic. Lower concentrations of all formulations demonstrated the absence of hemolysis and were therefore excluded from Figure 6 for clarity.

### 2.6 Liposomes Show Uninhibited Cell Interaction of Nanobody With HER2 Overexpressing Cells

**FIGURE 7:**
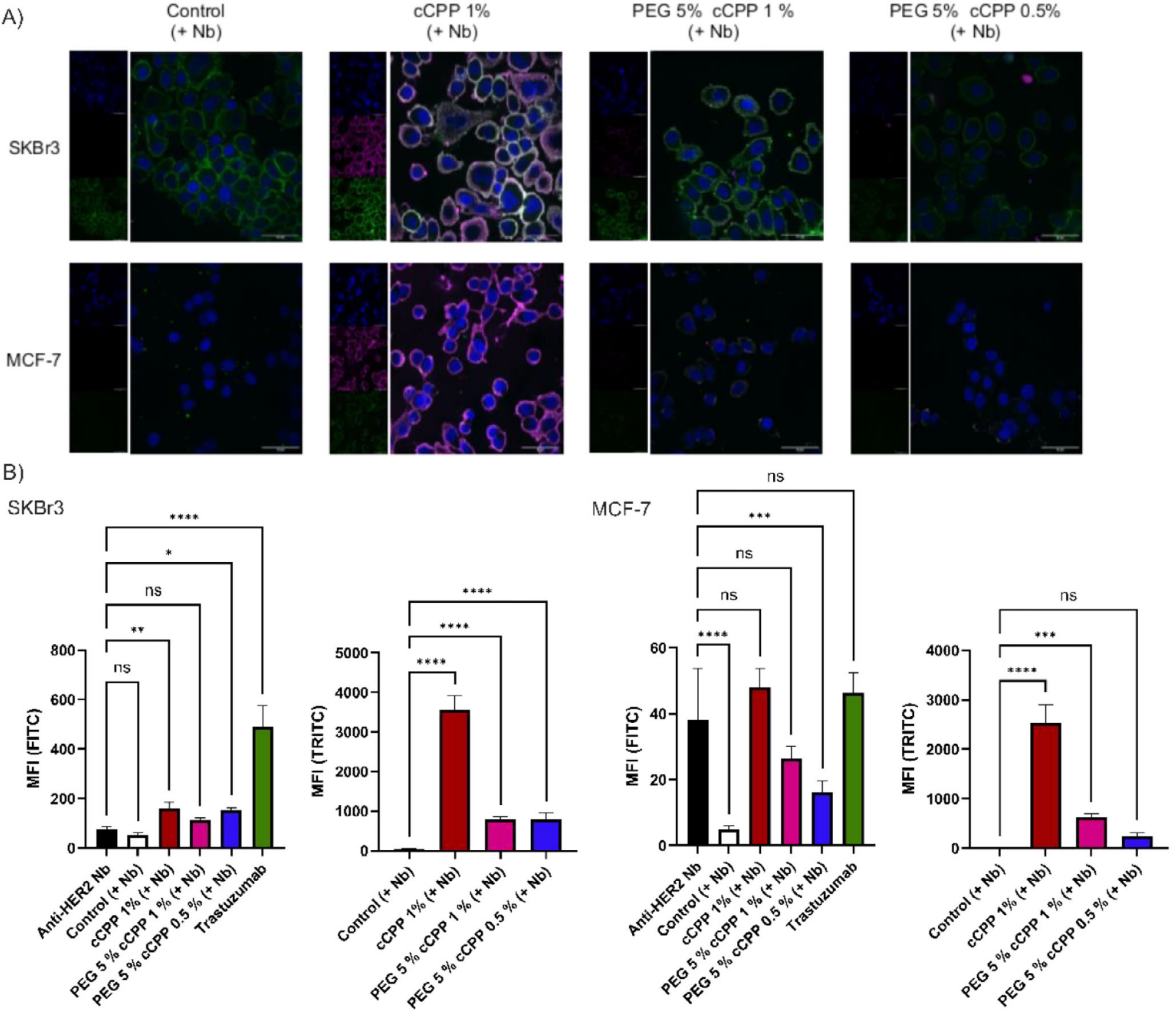
Qualitative and quantitative confocal analysis reveals formulation‐dependent cellular uptake. (A) Representative confocal micrographs showing the interaction of anti‐HER2 Nb‐loaded liposomes (5 µm Nb) with SKBr3 and MCF‐7 cells. The single channels (Green FITC: Atto488‐labeled Nb; Red TRITC: rhodamine‐labeled Carrier; Blue DAPI: Hoechst‐stained Nuclei) are displayed alongside the merged overlay. Scale bars = 50 µm. (B) Quantitative analysis confirms the visual observations. On HER2^+^ SKBr3 cells, the fluorescence intensity of the encapsulated Nb was comparable to or significantly higher than the free Nb. In contrast, carrier interaction was negligible for the unmodified control but pronounced for all surface‐modified formulations. On MCF‐7 cells, the Nb signal remained significantly lower compared to SKBr3 cells, while the carrier uptake profile was similar between cell lines. Data are presented as mean ± SD. Quantification was performed on a total of n = 15–20 cells per condition (5 randomly selected cells from 3–4 independent fields of view). Statistical significance was determined by one‐way ANOVA followed by Dunnett's multiple comparison test (^****^ *p* < 0.0001, ^***^ *p* < 0.001, ^**^ *p* < 0.01, ^*^ *p* < 0.1, *ns* = *p* > 0.05).

The cellular interaction of liposomes encapsulating anti‐HER2 Nb was assessed on SKBr3 and MCF‐7 cells. To ensure experimental consistency, the same liposome batch was used for both cell lines, and all formulations contained the same Nb concentration as the free Nb control (5 µm, see Figure 2). Figure 7 presents confocal microscopy images, displaying individual channels on the left and merged images on the right. The anti‐HER2 Nb is visualized in the green FITC channel, the liposomes in the red TRITC channel, and cell nuclei in the blue DAPI channel. Visual inspection of the confocal images reveals a green Nb signal for all formulations on SKBr3 cells. Conversely, on MCF‐7 cells, the Nb signal was only visually detectable in the cCPP 1% formulation. Regarding the liposomal carrier (red signal), intensity appeared negligible for unmodified controls and faint for PEG‐lipid cCPP‐lipid formulations, but was clearly prominent in the cCPP 1% formulation across both cell lines. These qualitative visual observations are supported by quantitative analysis of the mean fluorescence intensity (MFI) (Figure 7B). The MFI analysis confirms the trends observed in microscopy, comparing the levels of liposomal Nb against the free Nb control and trastuzumab, while also quantifying the fluorescence intensity of the liposomal carriers themselves. For SKBr3 cells, trastuzumab showed the highest cell binding, which was also significantly higher (*p* < 0.001) than the free Nb. However, for the liposomal formulations, this trend was less pronounced. While the difference to the control was not significant for PEG 5% cCPP 1% (*p* > 0.05), it was significant for PEG 5% cCPP 0.5% (*p* < 0.05) and highly significant for cCPP 1% (*p* < 0.01). Still, in all cases, the liposomal formulations showed a higher Nb signal than the free Nb. The cCPP 1% formulation, which showed the highest Nb signal among all liposomal formulations, also exhibited the highest carrier signal across all formulations. Here, the effect of the included cCPP‐lipid increased the signal for all formulations significantly (*p* < 0.001) compared to the unmodified control liposomes. The control liposomes showed no measurable interaction and confirmed, therefore the visually observed absence of TRITC signal. For MCF‐7 cells, all measured FITC intensities were significantly lower than on the HER2^+^ SKBr3 cells. The minimal binding that could be observed with the MFI is nonsignificant compared to the free anti‐HER2 Nb and trastuzumab as well as for anti‐HER2 Nb and liposomal formulations. The significance only occurs when compared to liposomal formulations, which exhibit a lower MFI than the free Nb (control, PEG 5% cCPP 1%), conversely to the SKBr3 cells, where the liposomes showed slightly enhanced Nb interaction with the cells. For the TRITC signal, the pattern on MCF‐7 cells is very similar to the observed signal on SKBr3 cells, with the only exception being that PEG 5% cCPP 0.5% shows nonsignificant higher interaction than the control liposomes.

**FIGURE 8:**
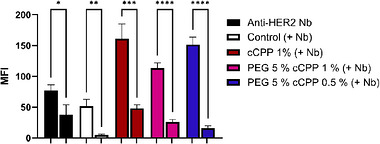
Quantitative comparison confirms HER2‐dependent cellular uptake. The mean fluorescence intensity (MFI) of the Nb signal is directly compared between HER2^+^ SKBR3 cells (left columns) and HER2–MCF‐7 cells (right columns) for each treatment. While the free Nb showed higher receptor binding on SKBR3 cells, this HER2‐specific accumulation was highly consistent and significant for all liposomal formulations (PEG 5% cCPP 1%, PEG 5% cCPP 0.5%, and cCPP 1%). In all cases, SKBR3 cells exhibited markedly higher intensities compared to the MCF‐7 control. Data are presented as mean ± SD. Quantification was performed on a total of n = 15–20 cells per condition (5 randomly selected cells from 3–4 independent fields of view). Due to unequal variances in the free Nb group, statistical significance was determined by Brown‐Forsythe and Welch ANOVA followed by Dunnett's T3 multiple comparison test (^****^ *p* < 0.0001, ^***^ *p* < 0.001, ^**^ *p* < 0.01, ^*^ *p* < 0.1).

To determine the difference between the Nb signal on SKBr3 cells and on MCF‐7 cells, the FITC‐MFI of all formulations on SKBr3 is depicted in Figure 8 and compared to the corresponding MFI on MCF‐7 cells, where the left column shows SKBr3 cells and the right column MCF‐7 cells. The difference in MFI is highly significant for all Nb and Nb‐liposomal treatments, although there is no targeting moiety on the surface of the liposomes.

### 2.7 Biodistribution and Macrophage Uptake in Zebrafish Larvae

To evaluate circulation time and immune evasion, zebrafish larvae of the Tg(kdrl: EGFP) (vascular endothelium) and Tg(mpeg1:Gal4:UAS: Kaede) (macrophages) transgenic lines were utilized.

**FIGURE 9:**
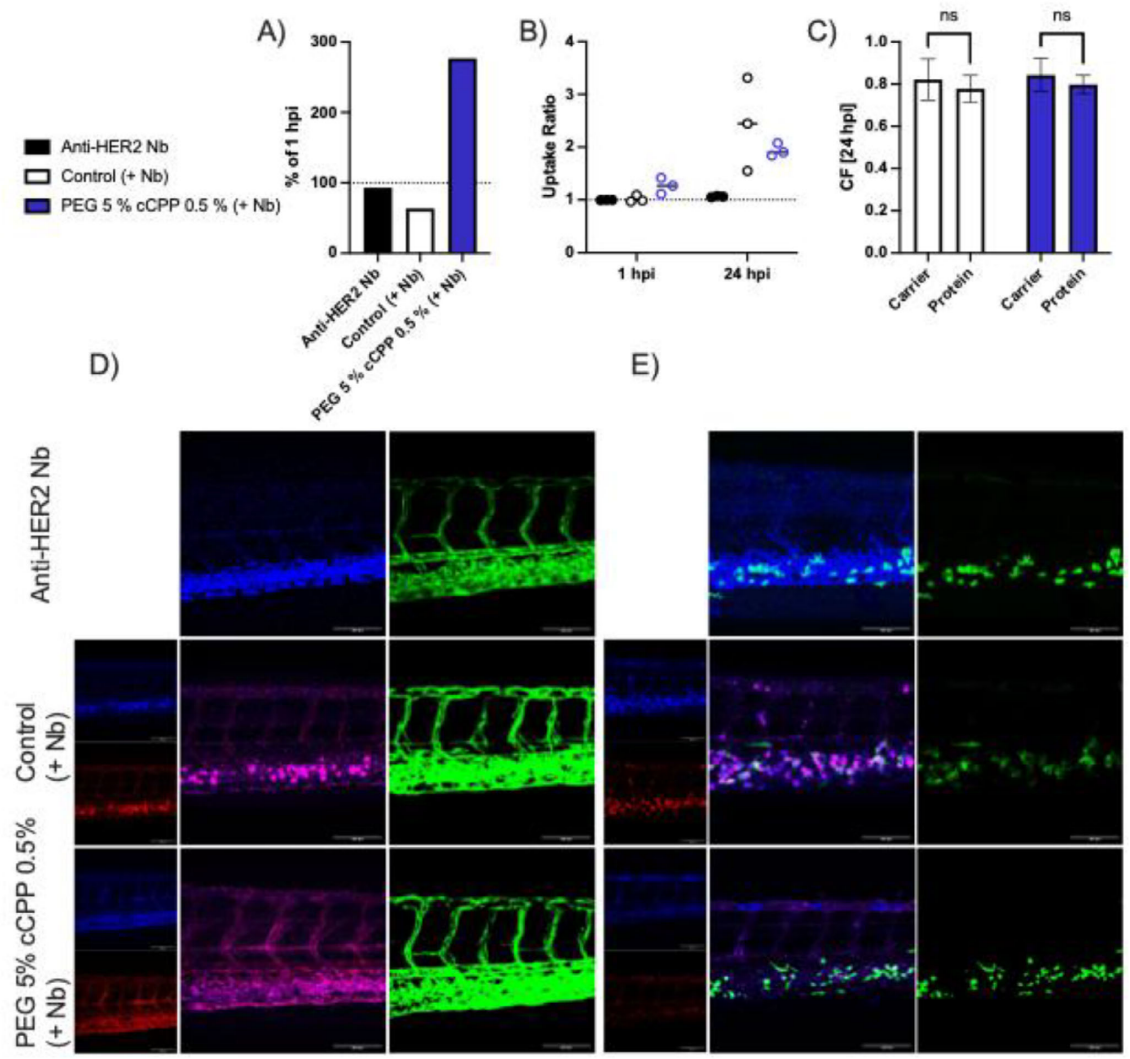
Zebrafish analysis reveals prolonged circulation and immune evasion of the PEG 5% cCPP 0.5% formulation. (A) Quantification of mean fluorescence intensity (MFI) in the vasculature of Tg(kdrl: EGFP) larvae. Data at 24 h post‐injection (hpi) are normalized to 1 hpi (set to 100%). While the control formulation shows a decrease in circulation, the PEG 5% cCPP 0.5% formulation exhibits increased retention, significantly higher than the free Nb. (B) Dot plot analysis of macrophage uptake in Tg(mpeg1:Gal4:UAS: Kaede) larvae. The free Nb shows no uptake, and the PEG 5% cCPP 0.5% formulation displays only slight interaction. In contrast, the control formulation shows significant uptake with high biological variance, confirming the stealth properties of the surface modification. (C) Analysis of the circulation factor (CF), comparing the ratio of encapsulated protein to carrier signal at 24 hpi. Non‐significant changes indicate that the liposomal carrier systems remain intact and stable in circulation after 24 h. (D) Representative confocal microscopy of Tg(kdrl: EGFP) larvae (endothelial cells marked in green) injected with the indicated substances (Red TRITC channel: rhodamine‐labeled Carrier; far‐red channel (pseudo colored blue): Atto647N‐labeled Nb; Middle: Merged). (E) Representative confocal microscopy of Tg(mpeg1:Gal4:UAS: Kaede) larvae (macrophages marked in green) injected with the indicated substances. Artefacts due to autofluorescence of pigment cells have been removed manually from the green channel. Data are presented as mean ± SD. Statistical significance was determined by a two‐way ANOVA followed by Sidak's multiple comparison test (*ns* = *p* > 0.05). Scale bars = 100 µm.

To evaluate circulation time, zebrafish larvae of the Tg(kdrl: EGFP) transgenic line were injected with the formulations. Quantitative analysis of the MFI within the vasculature at 24 h post‐injection (hpi), normalized to 1 hpi, reveals distinct profiles (Figure 9A). The free Nb remained at 93% of the initial intensity. The unmodified control liposomal formulation decreased to 64%. The modified PEG 5% cCPP 0.5% formulation (treatment) showed an increase in signal intensity to 276%. Confocal microscopy analysis (Figure 9D) shows that the free Nb and the PEG 5% cCPP 0.5% formulation were detectable throughout the vasculature at 24 hpi. In contrast, the control formulation was reduced in dorsal vessels, and localized signals were observed in the caudal vein plexus (CVP).

To quantify interaction with the innate immune system, the uptake ratio (intracellular/extracellular signal) was analyzed in Tg(mpeg1:Gal4:UAS: Kaede) larvae (Figure 9B). At 1 hpi, all groups showed a baseline ratio of approximately 1.0. At 24 hpi, the free Nb remained at baseline levels. The control formulation exhibited an uptake ratio ranging from 1.5 to 3.3, and the PEG 5% cCPP 0.5% formulation showed a mean uptake ratio of 1.8 with lower variance compared to the control group. In the confocal images (Figure 9E), macrophages in the CVP displayed colocalization with the control formulation. Macrophages in the treatment group showed limited colocalization, with the formulation signal primarily located in the extracellular space.

To assess the integrity of the formulation, the circulation factor (CF) of the carrier (red channel) and the protein cargo (blue channel) were compared at 24 hpi. No significant difference was observed between the CF of the carrier and the protein in either of the control and treatment formulations (Figure 9C). Confocal imaging confirmed the spatial overlap (colocalization) of the carrier and protein signals in the vasculature.

### 2.8 Pharmacokinetic Studies in Rats Demonstrate Organ Distribution Alteration Compared to Free Nanobody

**FIGURE 10:**
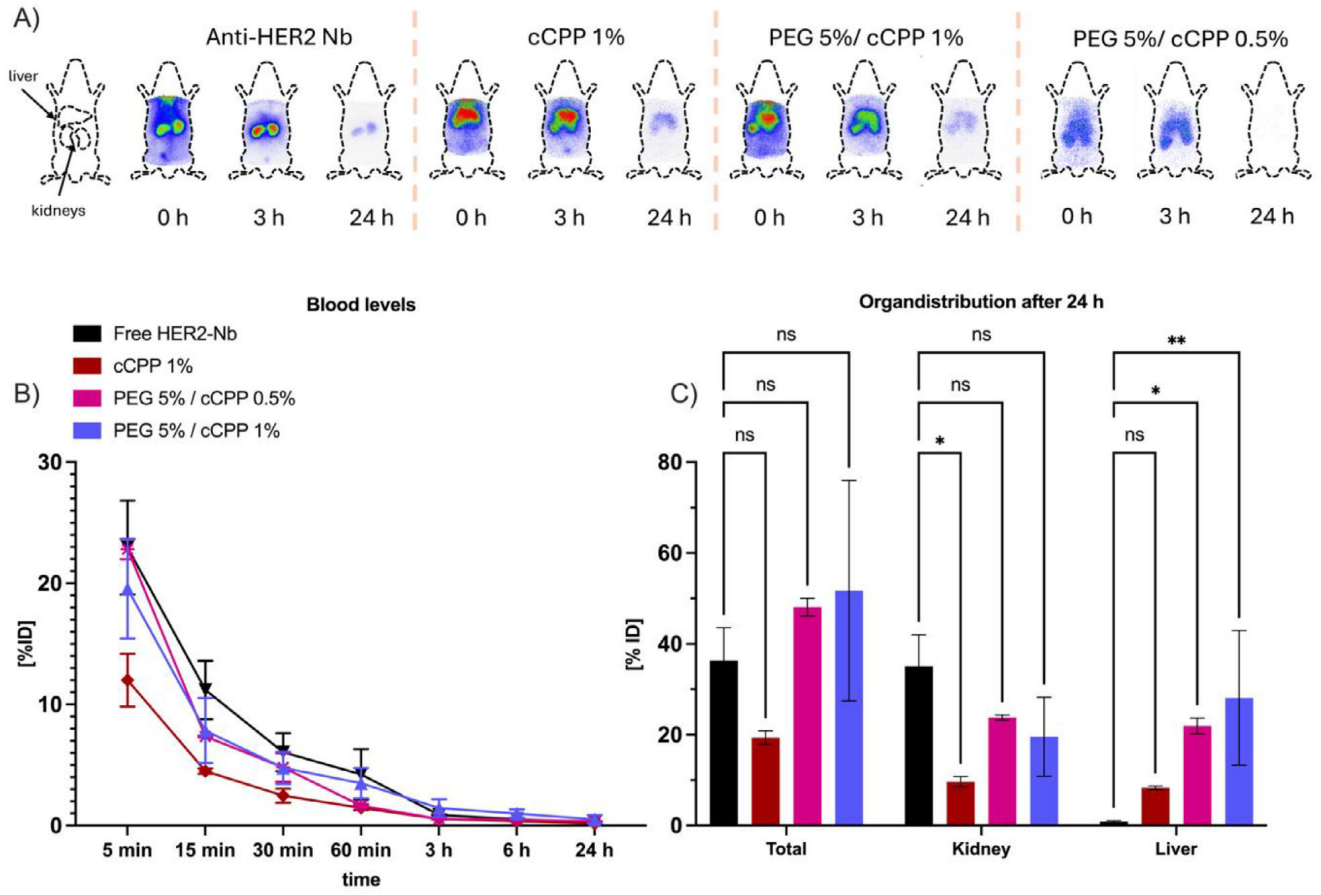
Altered biodistribution profiles of liposomal formulations. (A) Representative, qualitative, whole‐body scintigraphic images of Wistar rats injected with ^99m^Tc‐labeled anti‐HER2 Nb, either free or encapsulated in liposomes. Images were acquired immediately 0, 3, and 24 h post‐injection (p.i.). While the free Nb shows rapid renal clearance, the liposomal formulations display a distinct biodistribution pattern characterized by predominant accumulation in the liver. *Note*: Scintigraphic images represent a qualitative assessment. The visually lower intensity in the PEG 5% cCPP 0.5% representative image is a technical artifact resulting from the use of a thicker collimator, which was required to prevent detector saturation due to a higher absolute administered radioactive dose in this specific animal. (B) Blood levels and (C) organ distribution of Nb formulations. Blood levels, displayed as percentage of injected dose (%ID), show rapid clearance from the bloodstream for all formulations. Organ distribution 24 h post‐administration (%ID) demonstrating the accumulation of formulations in the liver and kidneys. While the total retained dose of liposomal formulations and free Nb does not differ significantly, the tissue distribution shows significantly reduced kidney uptake and increased liver accumulation for the liposomal formulations. Statistical significance was determined by a two‐way ANOVA followed by Tukey's multiple comparison test (^**^ *p* < 0.01, ^*^ *p* < 0.1, *ns* = *p* > 0.05).

Following the zebrafish studies, the biodistribution of free anti‐HER2 Nb and liposome‐encapsulated Nb was evaluated in healthy Wistar rats. As an initial qualitative assessment, in vivo scintigraphic imaging of a dedicated cohort over 24 h (Figure 10) revealed distinct distribution profiles. While the free Nb accumulated exclusively in the kidneys, the liposomal formulations accumulated primarily in the liver. The hepatic signal intensity correlated positively with the amount of non‐masked cCPP. Over the 24‐h course, this hepatic signal declined, while a concurrent signal emerged in the kidneys, resulting in a balanced distribution between the two organs at this endpoint.

Blood clearance profiles (Figure 10) indicated rapid extravasation for all formulations (as percentage of the injected dose, %ID). Quantitative biodistribution analysis at 24 hpi was restricted to the kidneys, liver, and blood, as uptake in non‐clearance tissues was negligible. In contrast to the free Nb control (35.04%ID), the cCPP‐modified formulation demonstrated significantly reduced renal accumulation (9.65%ID). The inclusion of PEG‐lipids further modulated this retention profile.

## 3 Discussion

The anti‐HER2 Nb was successfully expressed in *E. coli*, resulting in the high yields required for subsequent formulation studies. Mass spectrometric analysis (Figure 1) confirmed the expected molecular weight as the most prominent peak, alongside a minor species exhibiting N‐terminal methionine retention. This incomplete excision of the N‐formylmethionine (fMet) start codon is a well‐known phenomenon in prokaryotic expression [23]. However, as the N‐terminus is located distal to the antigen‐binding loops (CDRs), this heterogeneity does not impact the functional properties of the Nb [24]. The expression of Nbs in *E. coli*, while being a cost‐effective approach, frequently leads to the formation of insoluble inclusion bodies [25, 26]. This necessitates an additional processing step involving the denaturation of the Nb to achieve solubility, followed by refolding to restore its native, functional conformation. The establishment of optimal refolding parameters is a critical and protein‐specific step for each recombinant construct. CD spectroscopy confirmed the correct secondary structure (Figure 1), which matched the mammalian‐expressed (CHO) reference Nb. The structural analysis was consistent with literature suggesting that Nbs primarily consist of two β‐sheets, forming the core β‐sandwich, where each sheet consists of four, respectively, five antiparallel β‐strands with the β‐turns connecting the strands [27]. While the spectra show small deviations between the *E. coli* and CHO‐cell expressed Nb, the overall shape remains consistent, suggesting that the protein is correctly folded in both cases with only minor conformational nuances. Following structural validation, cell binding was assessed on HER2^+^ SKBr3 cells and HER2– MCF‐7 controls using confocal microscopy and flow cytometry (Figure 2). Trastuzumab served as the clinical gold standard for comparison. The anti‐HER2 Nb demonstrated a membrane binding pattern and specificity comparable to trastuzumab, with a strong signal on SKBr3 cells and negligible binding to MCF‐7 cells. While trastuzumab exhibited higher fluorescence intensity, which is likely due to the avidity effects of the bivalent antibody compared to the monovalent Nb [28], the receptor specificity remained equivalent.

Following functional validation, the Nbs were encapsulated into liposomes to improve their pharmacokinetic profile. DC was utilized as a time‐ and cost‐effective technique known for producing small, uniform liposomes with high drug loading (Figure 3) [29]. This was supported by the Cryo‐TEM analysis, which revealed small and homogenous formulations. Small differences in size and morphology are commonly known as freezing artefacts and are negligible [30, 31]. The morphological differences and the quantitative size increase observed between the unmodified control (approx. 98 nm) and the dual‐modified formulations (approx. 135 nm) raise the important question of how these physical changes influence in vivo circulation and tissue accumulation. In nanomedicine, carrier size is a critical determinant of biodistribution, with a well‐established physiological size threshold of approximately 200 nm for mechanical filtration by the interendothelial slits of the spleen [32]. Since both the unmodified and the functionally modified liposomes remain well below this critical 200 nm threshold, the size increase itself is unlikely to be the primary driver of their altered pharmacokinetic profiles. Instead, the size increase is a natural biophysical consequence of the applied surface modifications, specifically the steric hydration shell of PEG and the electrostatic repulsion of the cCPPs.

Although encapsulating macromolecules like biologics in liposomes is often associated with drug loading challenges, the DC technique enabled high Nb EEs across all formulations. Unmodified control liposomes exhibited the highest EE. While the inclusion of PEG‐lipids led to a reduction in EE, the co‐incorporation of cCPP‐lipids effectively restored encapsulation levels. This can be attributed to electrostatic interactions. Given the Nb's pI of 6.2, the protein carries a net negative charge in PBS, which promotes interaction with the cationic cCPP‐lipid, possibly enhancing encapsulation. Conversely, the steric hindrance imposed by the PEG‐chains likely limits this capacity in PEGylated formulations. Zeta potential measurements served as an indicator for surface charge stability and confirmed the successful insertion of the cCPP‐lipid. In formulations containing both cCPP and PEG‐lipids, the zeta potential was notably reduced compared to cCPP‐only samples. This effect is likely due to the steric shielding of the PEG‐chains. At 5 mol%, PEG is known to transition between mushroom and brush conformations [33], effectively masking the positive charge of the cCPP. This shielding is advantageous as it may reduce nonspecific adhesion to non‐target cells. Further, the significant size increase observed in modified formulations can be attributed to both the electrostatic repulsion between cationic lipids and the hydrodynamic volume added by the PEG layer.

The temperature‐dependent fluorescence spectroscopy of membrane‐embedded Laurdan showed broad thermotropic phase transitions for all formulations, which (Figure 4) is characteristic of cholesterol‐containing membranes, as cholesterol modulates membrane fluidity [34, 35]. The addition of PEG‐lipids did not further broaden the phase transition, as has been reported for membranes without cholesterol [36]. The GP analysis revealed a hysteresis for the cCPP 1% formulation (Figure 5). This may reflect either irreversible membrane alterations triggered by thermal stress under the experimental conditions or slowed relaxation kinetics of the thermally driven membrane reorganization due to the presence of cCPP. The increase in GP between consecutive heating scans indicates increased lipid order and may result from irreversible membrane reorganization in the presence of cCPP. However, the interplay between PEG‐lipid and cCPP‐lipid extends beyond surface interactions, significantly influencing the biophysical properties of the membrane. PEG‐lipids appeared to mitigate cCPP‐associated membrane destabilization, suggesting a stabilizing effect on membrane structure. This study provides the first biophysical characterization of membrane dynamics in such PEG‐ and cCPP‐dual‐modified liposomes. The GP analysis confirmed an increase in the lipid packing density of the modified formulations, while highlighting the stabilizing effect of the PEG‐lipid on cCPP‐functionalized bilayers.

In conclusion, this study demonstrates that while therapeutic proteins demand delivery systems precisely tuned to their physicochemical properties, this liposomal platform offers a robust and versatile technology.

To evaluate the biocompatibility of the developed formulations and rule out potential off‐target toxicity of the Nb itself, hemolysis and cytotoxicity assays were performed (Figure 6). None of the formulations nor the free Nb induced hemolysis (on human erythrocytes) or cytotoxic effects on kidney (HEK293) and liver (HepG2) model cell lines. Interestingly, cell viability values exceeding 100% were observed in liposome‐treated samples. This phenomenon is frequently reported in liposomal toxicity studies and is attributed to a metabolic boost rather than actual cell proliferation. The cellular uptake of lipids provides an additional energy source, enhancing mitochondrial activity and thus increasing the reduction of the PrestoBlue reagent [37]. This effect is particularly pronounced in cell lines with high endocytic turnover, such as HEK293. In conclusion, neither the formulations nor the Nb exhibited cytotoxicity toward blood, liver, or kidney cells, suggesting a favorable safety profile for potential in vivo administration.

While demonstrating a favorable biocompatibility profile is crucial, it is equally critical to ensure that the binding capacity of the Nb to the HER2 receptor is not compromised by the carrier. Therefore, confocal microscopy was used to assess the cellular interaction of the lipid carriers and the protein with HER2^+^ (SKBr3) and HER2– (MCF‐7) cells (Figure 7). Typically, liposomal carriers lacking specific surface ligands might be expected to show comparable, non‐specific interaction patterns in both cell lines. Contrary to this expectation, a substantial difference was observed. The Nb signal was significantly higher on SKBr3 cells compared to MCF‐7 cells across all formulations. These results suggest that the receptor selectivity was not only preserved upon encapsulation but was even more pronounced in the liposomal formulations. Further, liposomal packaging led to a preferential binding of the encapsulated Nb compared to the free Nb. These findings point toward a synergistic effect between the liposomal carrier and the cargo, the formulation appears to enhance target accumulation while non‐specific interactions with HER2– cells, which were slightly lower than those observed for the free Nb, were even reduced (Figure 8). The robust target engagement observed in the confocal microscopy analyses (Figure 7) raises an important mechanistic consideration regarding the interaction between the fully encapsulated Nb and the surface‐exposed HER2 receptors. Since an intact liposomal bilayer sterically prevents the encapsulated protein from binding to extracellular targets, the robust HER2‐signal strongly implies an active role of the CPPs in mediating local payload release. It is well‐documented that arginine‐rich CPPs initially tether nanocarriers to the plasma membrane via strong electrostatic interactions with heparan sulfate proteoglycans [38]. Following the membrane contact, CPPs are known to induce significant local lipid remodeling. We hypothesize that this membrane‐active property leads to a localized fusion or transient destabilization of the liposomal bilayer directly at the cell surface. Consequently, the liposomes function as reservoirs that undergo cCPP‐triggered opening, releasing a highly concentrated burst of the Nb payload into the immediate pericellular space. This mechanism ensures that a massive local concentration of the protein is generated right at the target site, allowing the Nbs to efficiently engage the HER2 receptors immediately upon carrier disassembly.

Given that PEGylation is a well‐established strategy to prolong circulation time and enhance immune evasion, the formulations were subsequently evaluated in a zebrafish larvae model to assess biodistribution and macrophage interaction (Figure 9). As expected, due to their small size, the free Nb showed negligible interaction with the innate immune system, remaining at baseline uptake levels. In contrast, the unmodified control formulation exhibited an increased macrophage uptake ratio characterized by high variance, which is consistent with a naturally heterogeneous immune response to foreign particles. The PEG 5% cCPP 0.5% treatment formulation, however, displayed a reduced uptake with minimal variance, suggesting a functional stealth effect. This immune evasion directly translated into the biodistribution profile observed at 24 hpi. The unmodified control liposomes were rapidly cleared from the circulation (MFI dropping to 64%) and primarily accumulated in the caudal vein plexus, a region associated with high macrophage activity, thereby further corroborating the quantitative uptake data. Conversely, the treatment formulation demonstrated a prolonged circulation time. Notably, the fluorescence intensity of the treatment formulation increased markedly to 276% after 24 h. This phenomenon is likely attributed to a de‐quenching effect. While the fluorophores are densely packed and self‐quenched upon injection, the release or relaxation of the carrier structure during prolonged circulation allows for diffusion and subsequent increased signal. Although the free Nb also exhibited a high retention (93%) and remained detectable throughout the vasculature, qualitative analysis revealed a less pronounced signal in the dorsal vessels compared to the treatment, pointing toward non‐specific endothelial binding rather than active circulation. Finally, the CF was calculated to assess cargo stability. A divergence in the CF of the carrier and Nb at 24 hpi would indicate leakage. However, the differences between carrier and Nb CF remained non‐significant from 1 to 24 hpi. This confirms that the cargo remains stably encapsulated, validating the system's potential for robust drug delivery. To translate the zebrafish to a mammalian model, the biodistribution was further analyzed in healthy Wistar rats (Figure 10). Here, distinct tissue distribution patterns emerged. The free Nb localized exclusively to the kidneys after 24 h, accounting for approximately 40% of the injected dose. This renal accumulation is characteristic for low molecular weight Nbs [39]. In contrast, all liposomal formulations exhibited hepatic accumulation, a distribution profile previously established for cCPP‐modified carriers [17].

Interestingly, while PEGylation attenuates the strong primary cellular interaction of cCPPs, the distribution ratio between liver and kidneys remained relatively constant (approximately 50/50) across the modified formulations, contrary to the expectation that varying cCPP and PEG ratios would shift this distribution. However, despite this consistent organ distribution pattern, the total retained dose at 24 h varied significantly: PEGylated liposomes demonstrated markedly higher total body retention compared to cCPP‐only liposomes, confirming the benefits of the steric shielding. In conclusion, the pharmacokinetic properties are no longer determined by the encapsulated protein but are dictated by the nanocarrier, thereby successfully circumventing the rapid renal clearance inherent to free biologics.

Conceptually, the strategy of utilizing vesicular nanocarriers to modulate the pharmacokinetic limitations of free biologics has been explored recently. For instance, similar approaches using niosomes, vesicles formulated from non‐ionic surfactants and cholesterol, have successfully demonstrated that encapsulation can modulate the pharmacokinetic properties of anti‐VEGF Nbs [38, 40]. However, to the best of our knowledge, this study presents the first successful encapsulation of Nbs into true, phospholipid‐based liposomes. Building upon the foundational proof‐of‐concept provided by niosomal systems, our liposomal platform introduces a higher level of functional complexity. By utilizing a phospholipid bilayer, we enable the stable, dual‐surface modification with both PEG for steric shielding and cCPPs for active, receptor‐mediated cellular engagement, a sophisticated bio‐interface that expands the utility of Nb‐carriers from mere half‐life extension toward active, targeted intracellular delivery.

## 4 Conclusion

This study successfully established a liposomal delivery system for anti‐HER2 Nbs, utilizing a dual‐modification strategy with PEG and cCPPs. The approach addresses the critical limitation of free Nbs—rapid renal clearance—by shifting the biodistribution profile away from the kidneys. The formulations maintained high biocompatibility and exhibited a favorable safety profile with no signs of immunogenicity in zebrafish larvae. Furthermore, despite the steric shielding by PEG, the cCPP‐modification ensured that the Nbs retained their ability to specifically bind to HER2^+^ cancer cells. Consequently, this platform suggests an effective strategy to enhance the therapeutic window of low‐molecular‐weight biologics.

## 5 Experimental Section

### 5.1 Cell Culture

HER2^+^ SKBr3 cells and HER2– MCF‐7 cells (DSMZ, Braunschweig, Germany) were cultured in high glucose DMEM (Thermo Fisher Scientific, Rockford, USA) with 10% FBS (Bio&Sell, Feucht, Germany) and 1% Penicillin/Streptomycin (Sigma–Aldrich, St. Louis, USA). For HEK293 cells (DSMZ, Braunschweig, Germany) the medium was additionally supplemented with 1% non‐essential amino acids (Thermo Fisher Scientific, Rockford, USA) and 1% sodium pyruvate (Thermo Fisher Scientific, Rockford, USA). HepG2 cells (DSMZ, Braunschweig, Germany) were cultured in RPMI medium with 10% FBS and 1% Penicillin/Streptomycin. All cells were incubated at 37°C and 5% CO_2_ and tested monthly for *mycoplasma* contamination.

### 5.2 Anti‐HER2 Nanobody Expression and Purification

*Escherichia coli* BL21 (DE3) (New England Biolabs, Ipswich, USA) harboring a pET expression plasmid encoding an anti‐HER2 Nb with an N‐terminal polyhistidine tag for purification and radiolabeling were used for protein production. Cultures were induced with 1 mm IPTG and incubated overnight at 37°C. Cell lysis was performed using BugBuster protein extraction reagent supplemented with Benzonase (1:4000) (Merck KGaA, Darmstadt, Germany). Lysates were clarified by centrifugation (15 000 rpm, 20 min, 4 °C), and the resulting pellet was washed with TRIS buffer (50 mm TRIS, 150 mm NaCl, pH 8.0). Inclusion bodies were solubilized in Inclusion Body Solubilization Reagent (Thermo Fisher Scientific, Rockford, USA) by resuspension and incubation for 45 min at 4°C, followed by a second centrifugation step (15 000 rpm, 20 min, 4°C). The solubilized protein in the supernatant was purified by immobilized metal affinity chromatography (IMAC) using Ni‐NTA agarose (Qiagen, Hilden, Germany). Protein purity was assessed by SDS‐PAGE. For refolding, purified Nb fractions were diluted dropwise into a tenfold volume of refolding buffer (50 mm TRIS, 150 mm NaCl, 0.4 m L‐arginine, 5 mm reduced glutathione (GSH), 1 mm oxidized glutathione (GSSG), 1 m urea). The mixture was incubated overnight at 4°C. Subsequently, buffer exchange and sample concentration were performed using 3 kDa Amicon centrifugal filters (Merck KGaA, Darmstadt, Germany). Protein concentration was assessed by DC protein assays (Bio‐Rad Laboratories, Hercules, USA) on 96‐well plates following the manufacturer's instructions.

### 5.3 Structural Determination of Anti‐HER2 Nanobody

The structure of in *E. Coli* produced anti‐HER2 Nb was compared to the structure of a homologous Nb that was expressed in CHO‐cells. This Nb was commercially obtained to have a structural control (Evitria, Zurich, Switzerland). The structure was assessed by circular dichroism using a J‐1700 CD spectrometer (JASCO, Easton, USA). Protein solutions were diluted to 1 mg/mL in PBS and analyzed in a 1 mm quartz cuvette (Hellma Analytics, Mühlheim, Germany) over a wavelength range of 190–260 nm.

### 5.4 Mass Spectrometry of Anti‐HER2 Nanobody

Mass spectrometry of the anti‐HER2 Nb was performed using a HPLC‐MS/MS system, consisting of an Agilent 1200 Series HPLC (Agilent Technologies, Santa Clara, USA) equipped with a MassPREP Micro Desalting column (2.1 × 5 mm, 20 µm, 1000 Å; Waters, Milford, USA) maintained at 25°C. The chromatographic separation was performed with the following gradient: 0–2 min, 100% 0.1% formic acid in water; 2–2.5 min, linear increase to 50% acetonitrile; 2.5–5 min, isocratic at 50% acetonitrile; 5–7 min, linear increase to 100% acetonitrile. The flow rate was 0.5 mL/min, with an injection amount of 1–5 µg. Mass spectrometry was carried out using a Q Exactive Plus Orbitrap mass spectrometer (Thermo Fisher Scientific, Rockford, USA). Full MS spectra were collected over an m/z range of 1800–5000 in positive ion mode, with an in‐source collision‐induced dissociation (CID) of 10 eV.

### 5.5 Fluorescence Labeling

For confocal microscopy, the Nb was fluorescently labeled with either Atto 488‐NHS ester for cell studies or Atto 647N‐maleimide for zebra fish larvae studies (Atto‐TEC, Siegen, Germany). The labeling was performed according to the manufacturer's instructions, and the degree of labeling (DOL) was determined by Mass spectrometry.

### 5.6 Radiolabeling

For radiolabeling of the Nbs, ^99m^Tc was used. His‐tags of recombinantly expressed proteins can form stable complexes with Tc(I)‐carbonyl species. Sodium pertechnetate (Na[^99m^Tc]TcO_4_^−^), eluted from a Tekcis ^99^Mo/^99m^Tc generator (Curium Pharma, Boston, USA.), can be converted to the desired tricarbonyl form [^99m^Tc(H_2_O)_3_(CO)_3_]^+^ through a one‐step reduction with NaBH_4_ and CO as coordinating ligand for stabilization of the Tc(I) center [41]. For convenient lab handling, the CRS Kit (Paul Scherrer Institute, Villlingen‐PSI, Switzerland) was used. The desired amount of TcO_4_^−^ was diluted in 1 mL of NaCl and injected into the vial under inert atmosphere conditions. The mixture was heated for 20 min at 95 °C and then cooled for 7 min. Afterward, the vial was vented and the cap removed. The pH was adjusted to 6.5 with 0.25 m phosphate buffer and 1 m HCl. The mixture was added to the protein and incubated for 1 h at 37°C. Unbound technetium and buffer components were removed and replaced with PBS by centrifugation with 3 kDa Amicon centrifugal filters. Purity was assessed by RP‐HPLC with a C18 column and a linear gradient from 100% H_2_O + 0.1% TFA to 100% acetonitrile + 0.1% TFA in 5 min.

### 5.7 Flow Cytometry Analysis of Anti‐HER2 Nanobody Binding

HER2^+^ SKBr3 and HER2– MCF‐7 cells were harvested at approximately 80% confluence using 0.5% Trypsin–EDTA (Sigma–Aldrich, St. Louis, USA). Cells were then washed twice with PBS, counted, and resuspended in PBS containing 1% BSA (Sigma–Aldrich, St. Louis, USA) to a concentration of 1 × 10^6^ cells/mL. Aliquots of 2 × 10^5^ cells were transferred into V‐bottom 96‐well plates (Greiner, Kremsmünster, Austria) for blocking.

Atto488‐labeled anti‐HER2 Nb and Atto488‐labeled trastuzumab were diluted in PBS + 1% BSA to a final concentration of 5 µm. Cells were incubated in duplicates with either the Nb or the trastuzumab solutions for 2 h at room temperature in the dark under continuous shaking. Unstained cells served as a control to assess background fluorescence.

Following incubation, cells were washed three times with PBS + 1% BSA and finally resuspended in 150 µL PBS. Samples were analyzed on a MACSQuant Analyzer 10 flow cytometer (Miltenyi Biotec, Bergisch Gladbach, Germany) using standard filter settings for 488 nm‐excitable fluorophores. Flow cytometric data was analyzed using FlowJo v10.10 (BD Biosciences, Ashland, USA). Data quality control was performed using the built‐in QC tool, and the flowAI plugin was used for data cleanup [42]. Irregularities in flow rate, signal stability, and dynamic range were removed from further analysis. Gating was conducted on the cleaned‐up dataset: a loose gate was drawn over the main cell population based on forward and side scatter parameters, followed by a gate in the FSC‐A versus FSC‐H channels to exclude doublets. The median fluorescence intensity (medianFI) of the resulting single‐cell population was then determined. The specific binding ratio was determined by dividing the background‐corrected median fluorescence intensity (ΔmedianFI) measured on HER2^+^ SKBr3 cells by the ΔmedianFI measured on HER2– MCF‐7 cells, as described in Equation S3.

### 5.8 Confocal Imaging of Anti‐HER2 Nanobody–Cell Interactions

SKBr3 cells and MCF‐7 cells were cultured on µ‐slide eight‐well chambers (Ibidi, Gräfelfing, Germany) at a density of 80000 cells per well. Prior to seeding, the wells were coated with 10 µg/mL poly‐D‐lysine (Thermo Fisher Scientific, Rockford, USA) for 20 min to promote cell adhesion. After removing the coating solution, cells were seeded and incubated overnight to allow for proper attachment. The following day, the culture medium was aspirated, and cells were blocked for 2 h at room temperature using Pierce Protein‐Free Blocking Buffer (Thermo Fisher Scientific, Rockford, USA). Fluorescently labeled compounds were diluted in the same blocking buffer and incubated with the cells for 2 h at room temperature. After incubation, cells were washed with PBS and subsequently fixed with 4% paraformaldehyde (PFA) for 20 min. Following fixation and an additional PBS wash, cell nuclei were stained with NucBlue (Thermo Fisher Scientific, Rockford, USA) for 20 min at room temperature. After a final wash with PBS, images were acquired using a Nikon Ti‐Crest X‐Light V3 spinning disc confocal (Nikon cooperation, Tokyo, Japan). The setup was equipped with a Plan Apo λ 60x oil immersion objective (NA 1.40). Excitation was performed using 405 nm and 488 nm laser lines (Lumencor Celesta Light Engine), and images were recorded using an Andor Zyla sCMOS camera (2048 × 2048 pixels) controlled by NIS‐Elements software. Image processing and quantitative analysis were performed using FIJI/ImageJ software v2.16.0. Images are shown as representative micrographs. For visual clarity and equitable comparison, the display range (minimum and maximum pixel values) for each fluorescence channel was determined by the sample with the highest measured signal intensity and applied uniformly and linearly across all corresponding experimental images. An exception was made for the trastuzumab condition: due to its notably higher binding affinity and subsequent signal saturation, the display range for this group was adjusted independently and linearly to preserve spatial information. All quantitative analyses were conducted using the raw, unprocessed 16‐bit image data in Fiji/ImageJ software v2.16.0.

### 5.9 Liposome Preparation

Liposomes were prepared using the thin‐film hydration technique with DC (Zentrimix 380 R, HettichLab, Tuttlingen, Germany), as described previously [43, 44]. Detailed methodology and parameters are provided in Table S2.

Control liposomes consisted of 90 mol% soybean phosphatidylcholine (SPC; Lipoid, Ludwigshafen, Germany) and 10 mol% cholesterol (Sigma–Aldrich, St. Louis, USA). For surface‐modified formulations, cCPP‐lipid and PEG‐lipid were incorporated, final compositions can be found in Table S2.

The PEG‐lipid employed was PE 18:0/18:0‐PEG 2000 (Lipoid, Ludwigshafen, Germany). The cCPP‐lipid consisted of a in house synthesized cyclic nonaarginine‐cysteine peptide coupled to DSPE‐PEG(2000)‐maleimide (Lipoid, Ludwigshafen, Germany) prior to liposome preparation as reported previously [43]. For fluorescent labeling, 0.2 mol% 18:1 Lissamine Rhodamine PE (Avanti Polar Lipids, Alabaster, USA) was included in the lipid mixture. To introduce additional shear forces, SiLibeads (Sigmund Lindner, Warmensteinach, Germany) were added in an amount five times greater than the total lipid weight (Table S2).

Liposomes were purified with a self‐cast Sephadex G‐50 Fine column (GE Healthcare Bio‐Sciences, Uppsala, Sweden), where fractions were eluted in 250 µl steps. Fractions with liposomes were pooled and used for the subsequent experiments.

### 5.10 Liposome Characterization

#### 5.10.1 Size and PDI

To determine the size and homogeneity of the liposomal formulations, the Z‐average and PDI were determined with a Zetasizer Ultra (Malvern Panalytical, Malvern, UK). Liposomes were diluted 1:1000 in PBS. Measurement was performed in polystyrene cuvettes in triplicates with each replicate consisting of 10 runs at 173° measurement angle. Further parameters are provided in Section S2. Statistical significance was assessed using a one‐way ANOVA followed by Dunnett's multiple comparison test against the control liposome group (^****^ *p* < 0.0001).

#### 5.10.2 Zeta Potential

The Zetasizer Ultra (Malvern Panalytical, Malvern, UK) was further used for zeta potential measurement. Here, liposomes were diluted 1:20 in potassium dihydrogen phosphate, 50 mm, pH 7.4, and measured in triplicates with a maximum run number of 20 in folded capillary zeta cells (Malvern Panalytical, Malvern, UK). Additional parameters are provided in Section S3. Statistical significance was assessed using a one‐way ANOVA followed by Dunnett's multiple comparison test against the control liposome group (^****^ *p* < 0.0001).

#### 5.10.3 Encapsulation Efficiency

Encapsulation efficiency was determined using radiolabeled Nbs. Initially, the total radioactivity of the unpurified liposome preparation was measured using an activmeter (NUVIA instruments GmbH, Dresden, Germany). The liposomes were subsequently purified, and the radioactivity of each eluted fraction was quantified. To account for residual radioactivity, measurements were also taken from the empty column, Eppendorf tube, and pipette tips; these values were subtracted from the initial total radioactivity. The encapsulation efficiency was calculated by summing the radioactivity of all fractions containing liposomes and dividing this by the corrected total radioactivity. Additionally, the sum of radioactivity detected in all fractions and residual materials was compared to the initial radioactivity to verify complete recovery. Statistical significance was assessed using a one‐way ANOVA followed by Dunnett's multiple comparison test against the control liposome group (^****^ *p* < 0.0001).

#### 5.10.4 Cryo‐TEM Analysis

To assess the lamellarity of the liposomal suspensions, cryogenic transmission electron microscopy (Cryo‐TEM) imaging was performed by Dr. Mohamed Chami at the BioEM Lab, Biocenter Basel, Switzerland, following their established protocols and as reported previously [17, 45]. 4 µL of the Nb‐containing liposomal formulations (10 mg/ml lipid concentration) were applied onto glow‐discharged holey carbon‐coated Lacey grids (Ted Pella, USA) and blotted with Whatman No. 1 filter paper. Vitrification was achieved by plunging the grids into liquid ethane cooled to −180°C using a Leica GP2 plunger (Leica Microsystems, Mannheim, Germany). The vitrified samples were then transferred to a Talos F200C electron microscope (FEI, Hillsboro, USA) equipped with a Gatan 626 cryo‐holder (Gatan, Pleasanton, USA) maintained at −175°C. Imaging was performed at an accelerating voltage of 200 kV under low‐dose conditions (40 e^−^/Å^2^). Micrographs were recorded using a 4K × 4K Ceta CMOS camera, resulting in a pixel size of 0.2 nm. Defocus values ranged from −2 to −3 µm.

#### 5.10.5 Laurdan Fluorescence Spectroscopy With Lipid State Observer

Biophysical properties of the lipid membranes were further characterized by using temperature dependent Laurdan fluorescence spectroscopy. To prepare Laurdan‐labeled liposomes, 0.5 mol% Laurdan was incorporated into the lipid films following the liposome preparation method described earlier. To ensure Laurdan incorporation did not affect liposome properties, the size, PDI, and zeta potential of Laurdan‐containing liposomes were measured and compared to those of unlabeled liposomes (S8). Liposomes were then diluted to a total lipid concentration of 1 mg/mL in PBS, and 280 µL of the suspension was transferred to a PCR tube (Greiner Bio‐One, Frickenhausen, Germany) containing a small magnetic stirrer. Measurements were performed using the LISO device, where a background spectrum of PBS was first recorded and automatically subtracted from the sample spectra. Samples were excited by an UV LED at 360 nm, and emission spectra from 390 to 800 nm were recorded. During three consecutive scans, samples were heated from −30°C to 60°C and cooled back to −30°C at a rate of 0.5 K/min. Prior to measurement, samples were cooled to −30°C to ensure thermal equilibrium. Temperature was monitored within the sample, and spectral resolution was set to 0.5 K. The detector's integration time was continuously adjusted for optimal signal quality. GP was calculated using the following formula, where *I(440)* and *I(490)* represent the measured fluorescence intensities at 440 and 490 nm, respectively.

(1) $$GP=\frac{I\left(440\right)-I\left(490\right)}{I\left(440\right)+I\left(490\right)}$$

The GP values were calculated from each emission spectrum and plotted as a function of temperature. The displayed data points were smoothed using a moving average over 9 consecutive measurements.

### 5.11 Cytotoxicity Assays

The biocompatibility of empty liposomes and free Nb were tested. Therefore, HEK293 and HepG2 cells were used to simulate the primary metabolizing organs, namely the kidneys and liver. In addition, all formulations and Nb were tested for hemolysis.

#### 5.11.1 PrestoBlue Cell Viability Assay

For the assay, cells were seeded in 96‐well plates (Greiner, Kremsmünster, Austria) at a density of 30 000 cells per well and incubated overnight to ensure proper attachment. For SKBr3 cells, plates were pre‐coated with poly‐d‐lysine as described above. Empty liposomes were diluted in the appropriate culture medium to a final concentration of 33.3 mm, representing higher concentrations than used in subsequent in vivo experiments. Anti‐HER2 Nb was diluted to 50 µm in the relevant culture medium. Pure culture medium was used as a positive control (100% viability), while 1% Triton X‐100 served as a negative control (0% viability). Cells were incubated with the respective treatments for 4 h at 37 °C, after which the plates were washed three times with PBS. PrestoBlue HS Cell Viability Reagent (Thermo Fisher Scientific, Rockford, USA) reagent was added according to the manufacturer's instructions, and cells were incubated for 45 min. Fluorescence was measured using an Infinite 200 Pro microplate reader (Tecan, Männedorf, Switzerland) with excitation at 540 nm and emission at 590 nm.

#### 5.11.2 Hemolysis

Blood from three healthy, consented human donors was collected into EDTA‐monovettes (SARSTEDT AG & Co. KG, Nümbrecht, Germany) Pooled blood was centrifuged at 2500 × g for 10 min at room temperature to remove plasma. Erythrocytes were washed three times with a double volume of PBS, followed by centrifugation at 2500 × g for 10 min after each wash. Liposomes and Anti‐HER2 Nb were prepared in PBS at starting concentrations of 25 mm (lipid) and 100µm, respectively, and serially diluted 1:1 in a V‐bottom 96‐well plate (Greiner, Kremsmünster, Austria), tested in technical duplicates. Liposomes could not be measured at higher concentrations due to their turbidity even after correction with a blank. PBS (0% hemolysis) and 20% (v/v) Triton X‐100 (100% hemolysis) served as controls. A final volume of 40 µL of the erythrocyte suspension was added to 40 µL of the test formulation in each well. Plates were incubated for 2 h at 37°C, followed by centrifugation at 2500×g for 10 min. The supernatant was transferred to a flat‐bottom plate, and hemoglobin release was quantified by measuring OD at 545 nm using a Sunrise reader (Tecan, Männedorf, Switzerland). Raw data were first corrected by subtracting the OD of corresponding formulation blanks (identical concentrations without erythrocytes) to account for turbidity. The resulting corrected values were then normalized by subtracting the OD of the PBS‐erythrocytes control. The percentage of hemolysis was finally calculated relative to the Triton X‐100 control.

### 5.12 Confocal Studies of Liposomal Formulations

For cell interaction studies of anti‐HER2 Nb‐loaded liposomes, Nb was labeled with Atto488 as previously described and incorporated into rhodamine‐labeled liposomes. Liposomes were purified according to the *liposome preparation* protocol, and the Nb concentration was determined. Treatments were then diluted in the appropriate cell culture media. Cells were prepared as described in the paragraph “*Nb binding on cells”* and liposomes were incubated for 2 h after blocking of the cells. Cells were fixed with 4% PFA. Cells were imaged using a Nikon Ti‐Crest X‐Light V3 spinning disc confocal. The setup was equipped with a Plan Apo λ 60x oil immersion objective (NA 1.40). Excitation was performed using 405, 488 and 546 nm laser lines (Lumencor Celesta Light Engine), and images were recorded using an Andor Zyla sCMOS camera (2048 × 2048 pixels) controlled by NIS‐Elements software. Image processing and quantitative analysis were performed using FIJI/ImageJ software v2.16.0. Images are shown as representative micrographs. For visual clarity and equitable comparison, the display range (minimum and maximum pixel values) for each fluorescence channel was determined by the sample with the highest measured signal intensity and applied uniformly and linearly across all corresponding experimental images. Data are presented as mean ± SD. Quantification was performed on a total of n = 15–20 cells per condition (5 randomly selected cells from 3–4 independent fields of view). Statistical significance was determined by one‐way ANOVA followed by Dunnett's multiple comparison test (^****^ *p* < 0.0001).

### 5.13 In Vivo Studies

#### 5.13.1 Zebra Fish Larvae

All zebrafish (*Danio rerio*) experiments were conducted in the Huwyler laboratory (Department of Pharmaceutical Technology, University of Basel, Switzerland) in compliance with Swiss animal welfare regulations. Fertilized eggs from adult transgenic zebrafish lines Tg(kdrl: EGFP) and Tg(mpeg1:Gal4:UAS: Kaede) were collected and incubated in standard E2 culture medium (5 mmol/L NaCl, 0.25 mmol/L KCl, 0.5 mmol MgSO_4_, 0.15 mmol/L KH_2_PO_4_, 0.05 mmol/L Na_2_HPO_4_, 0.5 mmol/L CaCl_2_, 0.71 mmol/L NaHCO_3_) complemented with phenyl‐2‐thiourea (PTU; 30 µg/mL; Sigma–Aldrich, St. Louis, USA) to inhibit melanization of zebrafish larvae (ZFL) at 28 °C. At 36 h post‐fertilization (hpf), eggs were manually dechorionated and screened for EGFP expression using a Leica MDG41 (Leica Microsystems, Mannheim, Germany) fluorescence microscope. At 48 hpf, ZFL were embedded in 0.3%(w/v) low‐melting agarose (BioRad Laboratoris; Hercules; USA), containing 0.01% Tricaine (MS‐222; Sigma–Aldrich, St. Louis, USA) in PTU‐supplemented E2 medium. Using a micromanipulator (Wagner Instrumentenbau, Schöffengrund, Germany) and a pneumatic Pico Pump PV830 (WPI, Sarasota, USA) under a Leica S8APO stereomicroscope (Leica Microsystems, Mannheim, Germany), 5 nL of Nb‐containing liposomes (20 mm lipid) or 5 nL of free Nb (2 mg/ml) were injected into the duct of Cuvier (DoC or common cardinal vein). For each fish line and formulation, 20 ZFL were injected. After injection, the larvae were incubated at 28°C, and viability was assessed 24 h post‐injection by visually confirming the presence of a heartbeat. Particle distribution was imaged at 1 h post‐injection (hpi) and 24 hpi by confocal scanning laser microscopy (CSLM) using an inverted Olympus FV3000 microscope (Olympus, Tokyo, Japan), equipped with either a 20× UPlanSAPO objective (NA 0.75) for whole‐larva imaging or a 30× UPlanSAPO oil immersion objective (NA 1.05) imaging of the tail region. Z‐stacks were acquired with 2 µm step sizes. Image processing and quantitative analysis were performed using FIJI/ImageJ software v2.16.0. Images are presented as representative micrographs of the maximum intensity projection of the z‐stacks. For visual clarity and equitable comparison, the display range (minimum and maximum pixel values) for each fluorescence channel was determined by the sample with the highest measured signal intensity and applied uniformly and linearly across all corresponding experimental images. Artefacts resulted from autofluorescence of pigment cells were removed manually from the images. For Tg(kdrl: EGFP) larvae, the circulation factor (CF) was calculated as follows:

(2) $$CF=\frac{mean\left(Intra\right)}{mean\left(Intra\right)+mean\left(Extra\right)}$$

For Tg(mpeg1:Gal4:UAS: Kaede) larvae, the enrichment ratio (R) was calculated as follows:

(3) $$R=\frac{mean\left(Intra\right)}{mean\left(Extra\right)}$$

Data are presented as mean ± SD. Statistical significance was determined by a two‐way ANOVA followed by Sidak's multiple comparison test (^****^ *p* < 0.0001).

#### 5.13.2 Rodent Studies

All experiments, performed in healthy female Wistar rats, were approved by the Animal Care and Use Committee at the governmental institution Regierungspräsidium Karlsruhe (Karlsruhe, Germany, reference number 35–9185.81/G‐199/21, date of approval: 23.11.2021), and all procedures were performed in accordance with institutional guidelines. Radioactive Nb was incorporated into liposomal formulations and purified as described above.

#### 5.13.3 Scintigraphic Imaging

Rats were anesthetized with isoflurane (Sedana Medical AG; Stockholm, Sweden), and the radioactive formulations were administered intravenously (∼300 µl) via the tail vein. Scintigraphic images were acquired at 0, 1, 3, 6, and 24 hpi using a γ‐camera (Biospace Lab, Paris, France).

#### 5.13.4 Blood Levels and Organ Distribution

For quantitative biodistribution analysis, an internal standard of each radioactive formulation was collected prior to injection and defined as the 100% reference. Under isoflurane anesthesia, rats received intravenous injections of the formulations via the tail vein. Syringes were weighed before and after administration to determine the exact injected dose (∼300 µl). Blood samples were collected at 5, 15, 30, 60, 180, and 360 min, as well as at 24 hpi. At 24 h, animals were sacrificed, dissected, and the kidneys, liver, and spleen were harvested. All tissue and blood samples were weighed, and radioactivity was quantified using a Cobra‐Auto γ‐counter (GMI, Ramsey, USA). The percentage of injected dose (%ID) for each sample was calculated from the measured radioactivity, the injected dose, the sample weight, and the body weight of the animal. For blood, total blood volume (BV) in milliliters was estimated using the formula [46]:

(4) BV=0.06×bodyweight(g)+0.77

Statistical significance was determined by a two‐way ANOVA followed by Tukey's multiple comparison test (^****^ *p* < 0.0001).

## Conflicts of Interest

The authors declare no conflicts of interest.

## Declaration of AI and LLM Use

During manuscript preparation, the authors used DeepL (Lingue) and Gemini Pro (Google) exclusively to improve language and readability. All parts were carefully revised and edited, and the authors take full responsibility for the content of this publication.

## Supporting information




**Supporting File**: advs75646‐sup‐0001‐SuppMat.docx.

## Data Availability

The data that support the findings of this study are available from the corresponding author upon reasonable request.
