# Supplementary material for: Balancing Stealth and Targetability: cCPP‐ and PEG‐Modified Liposomes for the Targeted Delivery of Anti‐HER2 Nanobodies
Source: Adv Sci (Weinh). 2026 May 11;13(43):e75646. doi: 10.1002/advs.75646 (PMC13336026; doi:10.1002/advs.75646)

**Table of contents**

**Title**

Balancing stealth and targetability: cCPP- and PEG-modified liposomes for the targeted delivery of anti-HER2 nanobodies

This study addresses the rapid renal clearance of anti-HER2 nanobodies by developing specialized liposomal carriers. It details the formulation of dual modified liposomes, demonstrating how PEGylation preserves structural integrity and cell-penetrating peptides enhance cellular interaction. Findings confirm maintained binding specificity in vitro, biocompatibility in zebrafish, and improved pharmacokinetic profiles in rats, offering a robust platform for enhancing low-molecular-weight biologics.

ToC figure


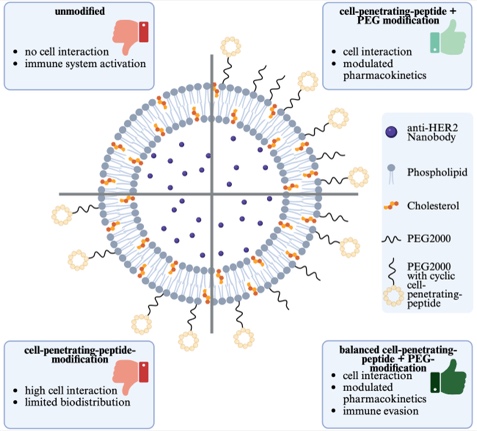


Supporting Information

Balancing stealth and targetability: cCPP- and PEG-modified liposomes for the targeted delivery of anti-HER2 nanobodies

Authors

*Giulia Pander, Maria del Pilar Palacios Cisneros, Clara Certa, Megan Stierli, Katharina Beck, Nicolas Färber, Lisa Blank, Fiona Tanner, Eric Mühlberg, Sabrina Wohlfart, Christian Kleist, Jörg Huwyler, Gert Fricker, Walter Mier, Philipp Uhl**

Figure S1: SDS-PAGE of purified anti-HER2 Nb

**Figure S1.** SDS-PAGE of purified anti-HER2 Nb. Marker bands (M) are shown in the left and right lane, all eluted fractions (F1-19) are shown in the middle. All fractions show the desired band at approximately 17 kDa and no other bands.

Section S1. Additional information on the amino acid sequences of the in *E.coli* expressed Nb and CHO-cell Nb

*E. coli*

MGSSHHHHHH SSGENLYFQS GSEVQLVESG GSLVQPGGSL RLSCAASGFT FDDYAMSWVR QVPGKGLEWV SSINWSGTHT DYADSVKGRF TISRNNANNT LYLQMNSLKS EDTAVYYCAK NWRDAGTTWF EKSGSAGQGT QVTVSSGSEN LYFQSWSHPQ FEK*

CHO-cell

EVQLVESGGS LVQPGGSLRL SCAASGFTFD DYAMSWVRQV PGKGLEWVSS INWSGTHTDY ADSVKGRFTI SRNNANNTLY LQMNSLKSED TAVYYCAKNW RDAGTTWFEK SGSAGQGTQV TVSSGGGGSH HHHHH*

Figure S2. Confocal analysis of the Nb expressed in *E.coli* and CHO-cells.

**Figure S2. Confocal analysis of the Nb expressed in *E.coli* and CHO-cells.**

The Nb (green) was analyzed on HER**^+^** SKBr3 cells. Both expression systems yield a functional Nb with similar binding pattern and strength. (Scale bars= 50 µm).

Table S1: Flow cytometry

**Table S1.** Measured median fluorescence intensities of anti-HER2 Nb, trastuzumab and untreated cells for the cell lines SKBr3 and MCF-7.

| Sample | Cell type | Median fluorescence intensity |
| --- | --- | --- |
| anti-HER2 Nb | SKBr-3 | 11.5 |
| trastuzumab | SKBr-3 | 22.1 |
| untreated | SKBr-3 | 0.36 |
| anti-HER2 Nb | MCF-7 | 1.46 |
| trastuzumab | MCF-7 | 1.02 |
| untreated | MCF-7 | 0.21 |

Equation S1: Flow cytometry

(S1)

$$Ratio=\frac{Specific signal\left( SKBr3 \right)-untreated signal (SKBr3)}{Specific signal \left( MCF-7 \right)-untreated signal (MCF-7)}$$

Table S2: Liposome preparation

**Table S2.** Run times and added volumes for the dual centrifugation with the Zentrimix. Detailed composition and number of beads added for all formulations are shown. Rhodamine was added for confocal and zebrafish larvae studies.

| Step | Time [min] | Volume added [µl] | Process |
| --- | --- | --- | --- |
| 1 | 15 | 27 | 2500 rpm, DC |
| 2 | 5 | 90 | 2500 rpm, DC |
| 3 | 1 | 133 | Vortex |

|  | SPC [mol%] | Cholesterol [mol%] | PEG-lipid  [mol%] | cCPP-lipid [mol%] | Rhodamine [mol%] | Beads  [mg] |
| --- | --- | --- | --- | --- | --- | --- |
| Control | 90 | 10 | - | - | - | 92 |
| cCPP 1% | 89 | 10 | - | 1 | - | 95 |
| PEG 5%  cCPP 1% | 84 | 10 | 5 | 1 | - | 108 |
| PEG 5%  cCPP 0.5% | 84.5 | 10 | 5 | 0.5 | - | 105 |
| Control | 89.8 | 10 | - | - | 0.2 | 92 |
| cCPP 1% | 88.8 | 10 | - | 1 | 0.2 | 95 |
| PEG 5%  cCPP 1% | 83.8 | 10 | 5 | 1 | 0.2 | 108 |
| PEG 5%  cCPP 0.5% | 84.3 | 10 | 5 | 0.5 | 0.2 | 105 |

S2: Size and PDI parameters

Further parameters were equilibration time = 60 s, refractive index solvent = 1.335, viscosity = 1.02 mPa*s, temperature = 25 °C, dielectric constant = 78.5 F/m, f(κα) model = Smoluchowski.

S3: Zeta potential parameters

Additional parameters were equilibration time = 60 s, refractive index material = 1.45 refractive index solvent = 1.335, viscosity = 0.8872 mPa*s, temperature = 25 °C, dielectric constant = 78.5 F/m, f(κα) model = Smoluchowski.

Figure S3: Inclusion of Laurdan into liposomes


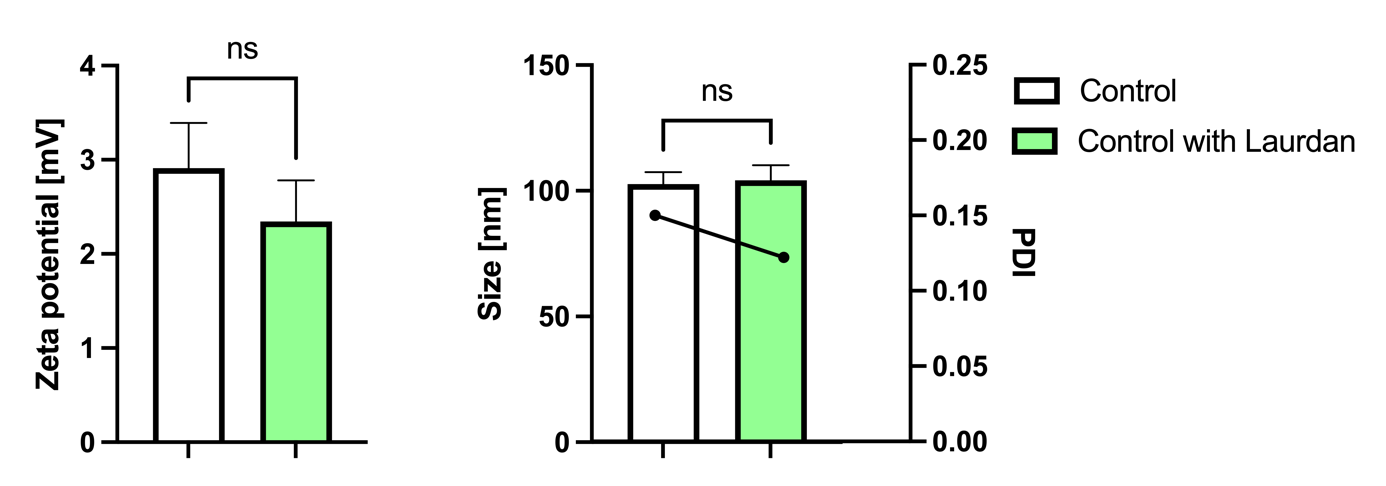


**Figure S3.** Comparison of liposomes with and without the addition of 0.5 mol% Laurdan. On the left the zeta potential shows a non-significant change between the two formulations. On the right the size is superimposed with the PDI. The formulations show non-significant differences. Statistical significance was determined by Welch’s t-test (****p < 0.0001).

Figure S4: 24-hour time-scan LISO


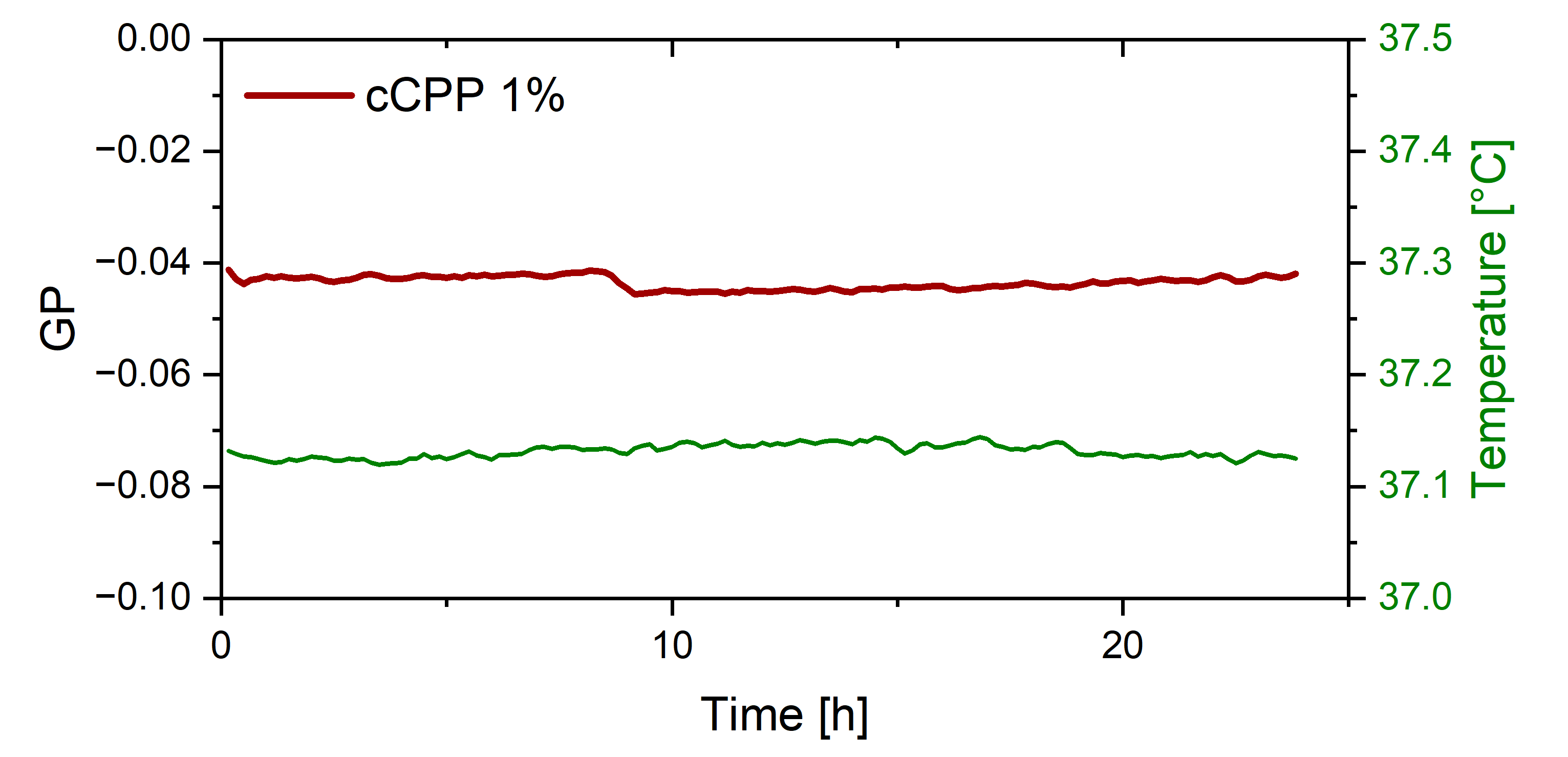


**Figure S4**. The 24-hour time scan of the cCPP 1% formulation in the LISO device. The left y axis shows the GP value, and the right y axis shows the temperature [°C]. The displayed data points are averages of three consecutive measurements, using a moving average filter.

**Raw confocal data for zebrafish Tg(mpeg1:Gal4:UAS:Kaede) larvae**

Pigment cells were removed manually and in accordance with the visually seen pigment cells in transmission view

**control formulation:**


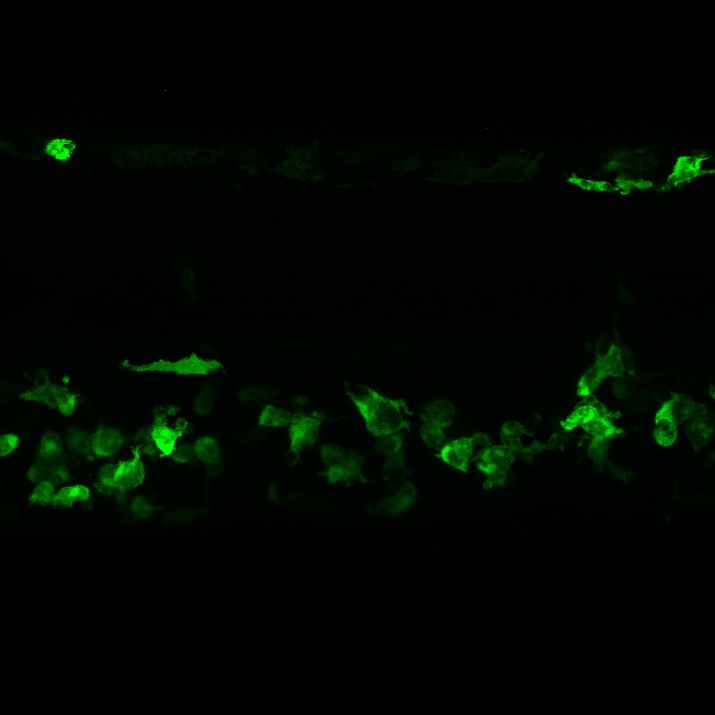

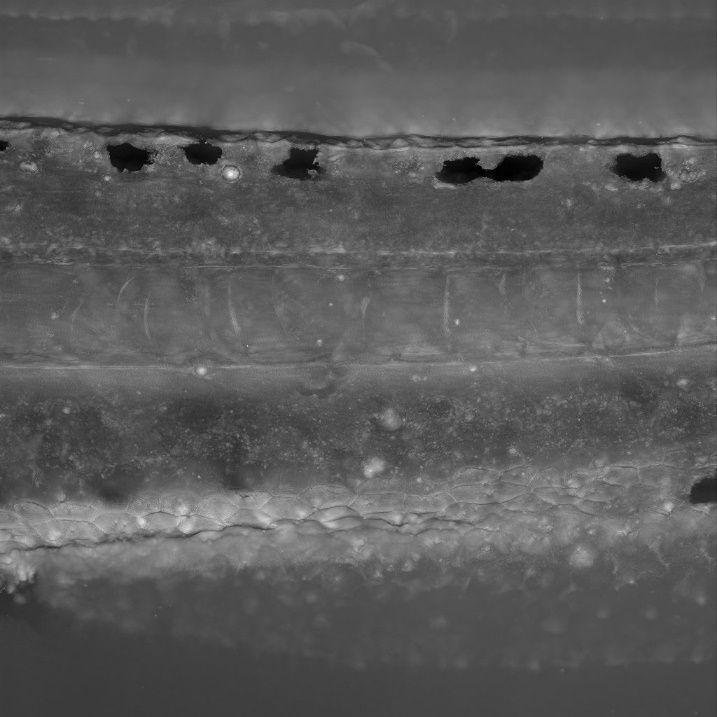


**PEG 5% cCPP 0.5%**


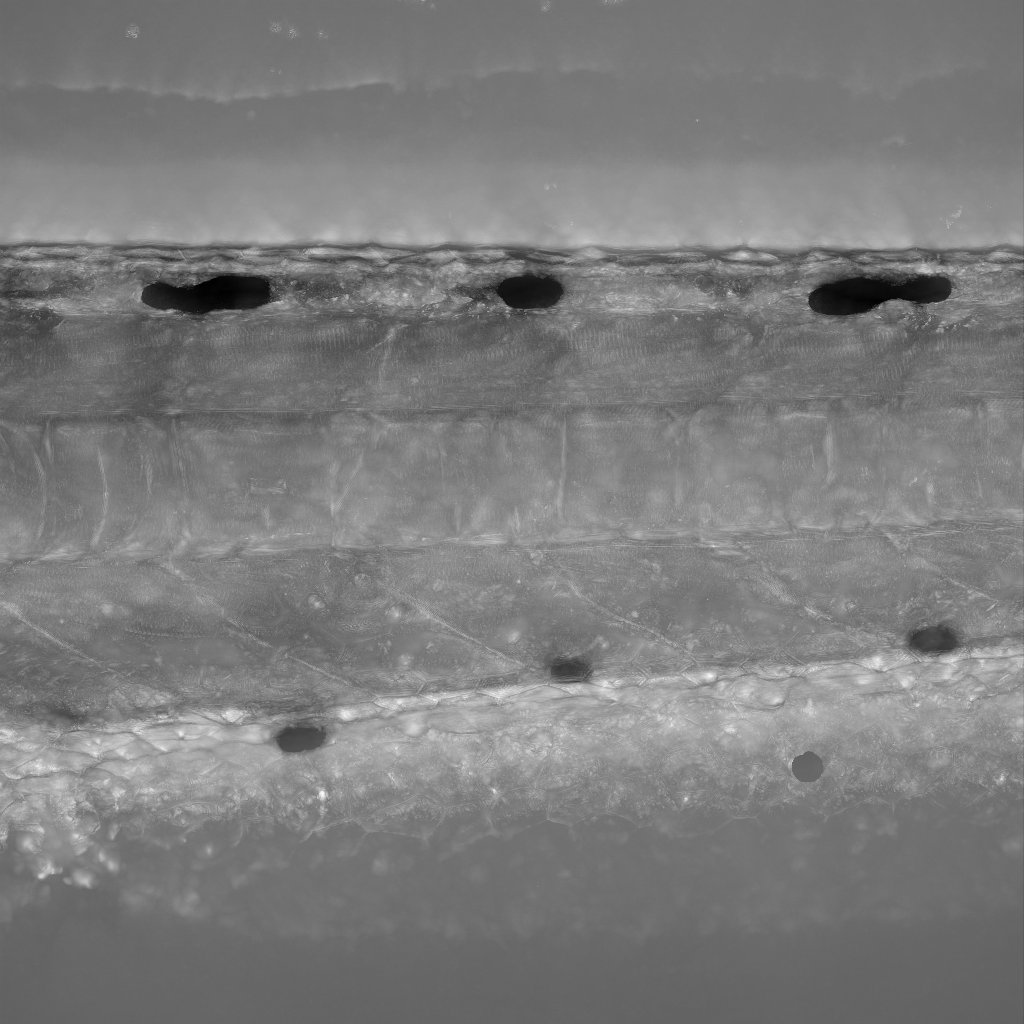

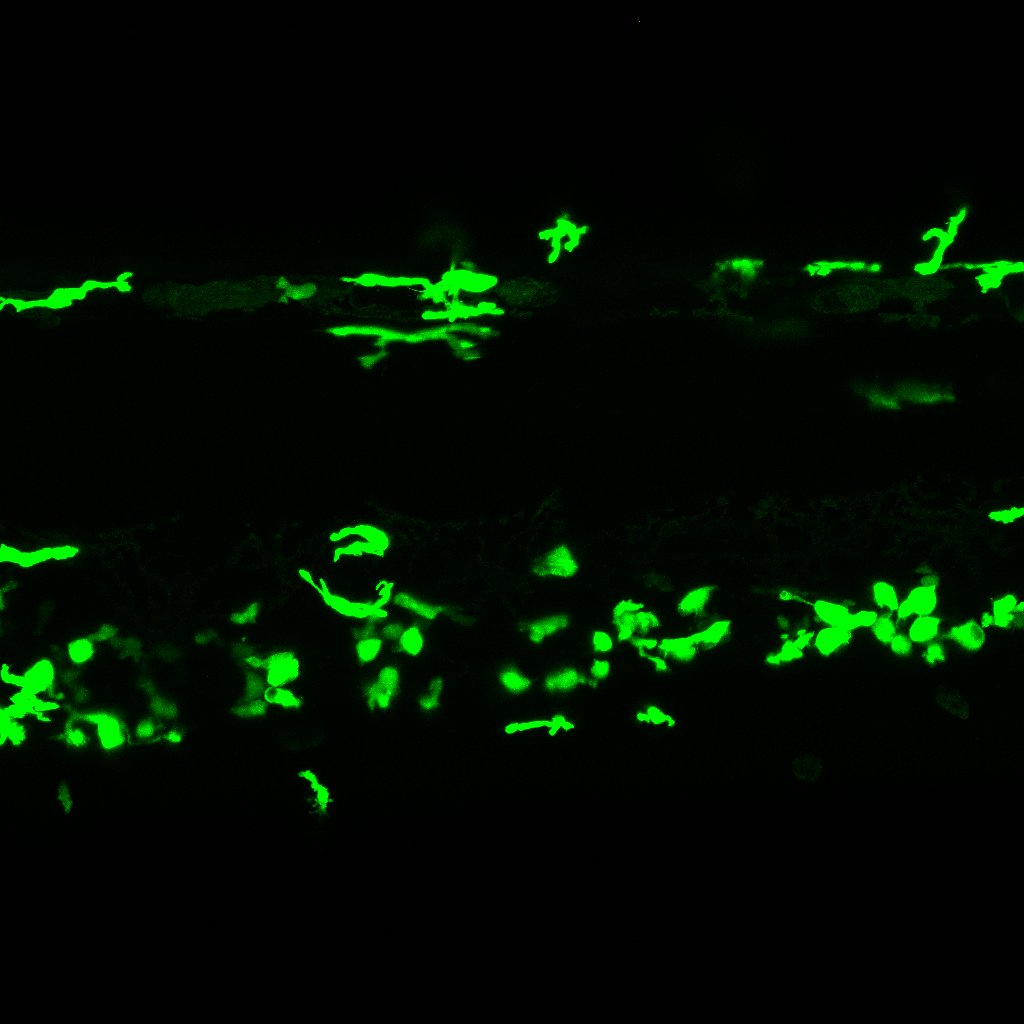


**free anti-HER2 Nb**


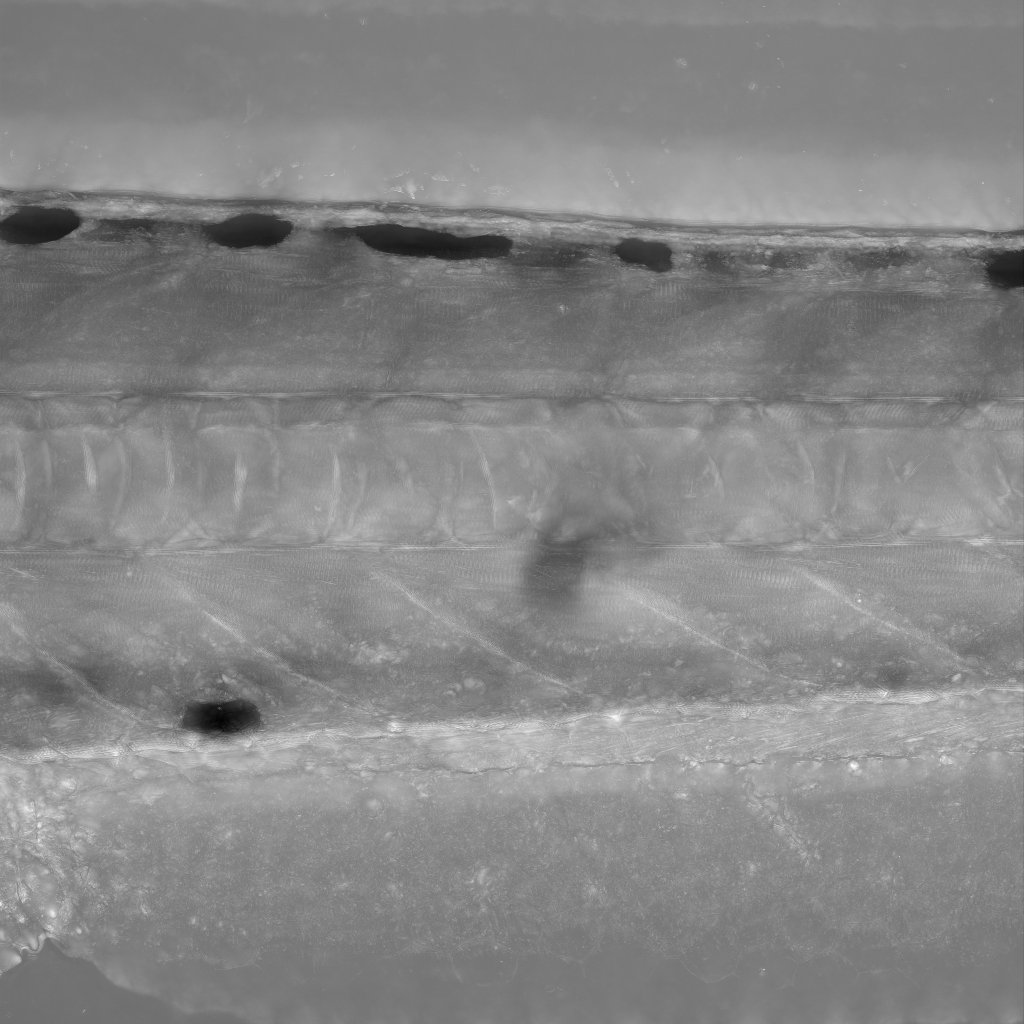

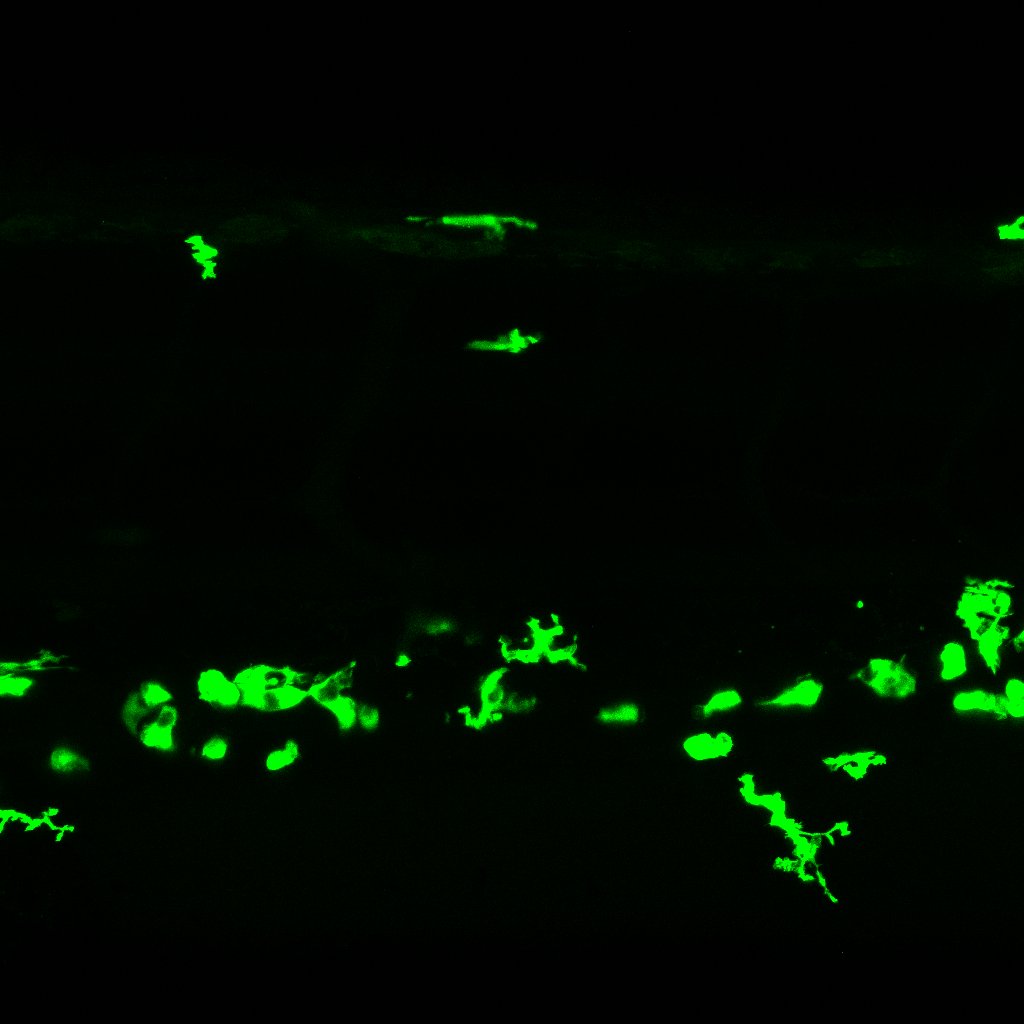

Supplement: Supplementary file 1 — Supporting File: advs75646‐sup‐0001‐SuppMat.docx. [file ADVS-13-e75646-s001.docx]
